# Associations between attainment of incentivized primary care indicators and incident lower limb amputation among those with type 2 diabetes: a population-based historical cohort study

**DOI:** 10.1136/bmjdrc-2020-002069

**Published:** 2021-04-26

**Authors:** Laura H Gunn, Eszter P Vamos, Azeem Majeed, Pasha Normahani, Usman Jaffer, German Molina, Jonathan Valabhji, Ailsa J McKay

**Affiliations:** 1 Department of Public Health Sciences, University of North Carolina at Charlotte, Charlotte, North Carolina, USA; 2 School of Data Science, University of North Carolina at Charlotte, Charlotte, North Carolina, USA; 3 Department of Primary Care and Public Health, Imperial College London, London, UK; 4 Imperial Vascular Unit, Imperial College London NHS Healthcare Trust, London, UK; 5 Department of Surgery and Cancer, Imperial College London, London, UK; 6 Division of Metabolism, Digestion & Reproduction, Faculty of Medicine, Imperial College London, London, UK; 7 Department of Diabetes and Endocrinology, St. Mary's Hospital, Imperial College Healthcare NHS Trust, London, UK; 8 NHS England and NHS Improvement, London, UK

**Keywords:** diabetic foot, primary healthcare, diabetes mellitus, type 2

## Abstract

**Introduction:**

England has invested considerably in diabetes care through such programs as the Quality and Outcomes Framework (QOF) and National Diabetes Audit (NDA). Associations between program indicators and clinical endpoints, such as amputation, remain unclear. We examined associations between primary care indicators and incident lower limb amputation.

**Research design and methods:**

This population-based retrospective cohort study, spanning 2010–2017, was comprised of adults in England with type 2 diabetes and no history of lower limb amputation. Exposures at baseline (2010–2011) were attainment of QOF glycated hemoglobin (HbA1c), blood pressure and total cholesterol indicators, and number of NDA processes completed. Propensity score matching was performed and multivariable Cox proportional hazards models, adjusting for disease-related, comorbidity, lifestyle, and sociodemographic factors, were fitted using matched samples for each exposure.

**Results:**

83 688 individuals from 330 English primary care practices were included. Mean follow-up was 3.9 (SD 2.0) years, and 521 (0.6%) minor or major amputations were observed (1.62 per 1000 person-years). HbA1c and cholesterol indicator attainment were associated with considerably lower risks of minor or major amputation (adjusted HRs; 95% CIs) 0.61 (0.49 to 0.74; p<0.0001) and 0.67 (0.53 to 0.86; p=0.0017), respectively). No evidence of association between blood pressure indicator attainment and amputation was observed (adjusted HR 0.88 (0.73 to 1.06; p=0.1891)). Substantially lower amputation rates were observed among those completing a greater number of NDA care processes (adjusted HRs 0.45 (0.24 to 0.83; p=0.0106), 0.67 (0.47 to 0.97; p=0.0319), and 0.38 (0.20 to 0.70; p=0.0022) for comparisons of 4–6 vs 0–3, 7–9 vs 0–3, and 7–9 vs 4–6 processes, respectively). Results for major-only amputations were similar for HbA1c and blood pressure, though cholesterol indicator attainment was non-significant.

**Conclusions:**

Comprehensive primary care-based secondary prevention may offer considerable protection against diabetes-related amputation. This has important implications for diabetes management and medical decision-making for patients, as well as type 2 diabetes quality improvement programs.

**Significance of this study**

**What is already known about this subject?**

- It is well established that diabetes-related lower limb amputations are highly preventable through comprehensive secondary prevention strategies.
- In England, national programmes incentivize primary care providers to complete associated care processes and meet related intermediate clinical outcomes. The specific indicators are based on available evidence, but their association with key clinical endpoints—including non-traumatic lower limb amputations—remains unclear.

**What are the new findings?**

- Our findings indicate that glycated hemoglobin (HbA1c) and cholesterol control levels incentivized by the English Quality and Outcomes Framework are associated with a lower risk of minor or major lower limb amputation. Additionally, HbA1c indicator attainment is associated with a lower risk of major-only lower limb amputation.
- Our findings also demonstrate that comprehensive provision of secondary prevention is associated with fewer minor or major amputations, as well as major-only amputations, and that there is scope to enhance provision of such care.

**How might these results change the focus of research or clinical practice?**

- Community-based secondary prevention has a vital role in preventing or delaying diabetes complications that have important implications for life expectancy and quality of life; thus, review of care implementation methods, including options to reduce the disutility associated with comprehensive care, may help reduce persistent gaps in care provision.

## Introduction

Lower limb amputations are an important and largely preventable complication of type 2 diabetes mellitus that impact considerably on the life expectancy and quality of life of those affected.^1–3^ In England, more than 9000 diabetes-related amputations are performed each year, with decreasing rates but higher absolute numbers of major amputations,^4^ and the annual direct healthcare costs of diabetic foot ulceration and amputation are approximately £1 billion.^5^ An estimated 27 465 diabetes-related amputations were carried out in England between 2015/2016 and 2017/2018, reflecting a 13.6% increase from the preceding 3-year period,^4^ and a frequency of amputation approximately 20 times higher than that observed in the population without diabetes.^1^ The considerable perioperative mortality risk associated with amputation is substantially elevated among those with diabetes.^6^ The particular level of risk depends on patient-related and surgery-related factors but typically exceeds 10%.^6 7^ Post-surgical 5-year mortality rates are also high (typically >50% among those with diabetes; and up to 90%–100% in some studies).^8 9^

Evidence suggests that many diabetes complications can be averted through high-quality and comprehensive secondary prevention.^10 11^ Therefore, over the past 20 years, England has adopted a number of national quality improvement strategies aiming to enhance community-based secondary prevention and reduce complication incidence. The Quality and Outcomes Framework (QOF)^12^ and the National Diabetes Audit (NDA)^13^ have been major components of this approach. The QOF represents one of the largest healthcare pay-for-performance schemes trialed worldwide. It has offered financial rewards to primary care providers who attain particular process and clinical care thresholds, including in diabetes care, since it was introduced in 2004 as part of a contract under which most general practitioners in England now operate. The NDA monitors provision of diabetes-related processes and intermediate clinical outcome attainment. It was introduced in 2003 and became required for primary care providers in 2017. The indicators monitored by QOF and the NDA are derived from related clinical guidelines that were, in turn, based on the associated evidence base. Indicators, therefore, overlap substantially. However, because the relevant evidence has often relied heavily on intermediate clinical outcomes, the indicators have relatively unclear associations with hard clinical endpoints, including amputation. Given the ongoing rise in numbers of diabetes-related lower limb amputations being performed in England,^1 4^ and the importance of this outcome to patients,^3^ understanding the relationships between the QOF and NDA indicators and amputation would help inform both diabetes management and medical decision-making discussions with individual patients and healthcare resource allocation, including for preventive activities. We, therefore, aimed to describe the associations between diabetes-related lower limb minor or major, as well as major-only, amputations and key QOF and NDA diabetes process and clinical indicators, among those with type 2 diabetes. The indicators of specific interest included the QOF glycated hemoglobin (HbA1c), blood pressure and cholesterol thresholds, and performance of the nine NDA care processes.

## Research design and methods

### Study design and data sources

The retrospective cohort was obtained from the UK Clinical Practice Research Datalink (CPRD) GOLD Database. The CPRD GOLD is a UK primary care database that contains longitudinal patient data from 1987 onwards. The data are collected during routine general practice activity. The database is representative of the UK primary care-registered population and currently includes roughly 50 million patients (16 million of whom are currently registered). For the majority of CPRD participants located in England, linked Hospital Episode Statistics (HES) and Office for National Statistics (ONS) mortality data are obtainable. The database has been used to study diabetes care processes and outcomes.^14 15^

Only participants with linked HES and ONS data were eligible for inclusion. They entered the cohort on April 1, 2010 so long as they had an existing type 2 diabetes diagnosis, were at least 18 years of age, had been registered with their practice for at least 1 year, and were not censored prior to April 1, 2011. Those with any existing minor or major amputation (as per our outcome definition below) prior to April 1, 2011, and those for whom a type 1 diabetes or other specified non-type 2 diabetes diagnosis was made at any point, were excluded. Those prescribed insulin within 3 months of a diabetes diagnosis if aged ≥35 years, or within 6 months of a diagnosis made if aged <35 years, were also excluded, as these may indicate inaccurate type 2 diabetes diagnoses.^16^ Cohort exit occurred at the earliest of: last CPRD data upload; transfer out of database; death; or December 31, 2017 (end of observational period). The code lists applied in cohort derivation and variable definitions (as below) are available in the online supplemental material.

10.1136/bmjdrc-2020-002069.supp1 Supplementary data

### Exposures

The exposures of interest included attainment of the QOF HbA1c (≤59 mmol/mol; 7.5%), blood pressure (≤140/80 mm Hg), and total cholesterol (≤5 mmol/L) indicators within the 2010–2011 financial year. Attainment was defined according to the QOF Business Rules V.38.0.^17^ The most recent measurements in the year of interest were used to determine indicator status, and an indicator ‘not met’ status was assigned where no measurement was made. A further exposure variable measuring implementation of NDA annual care processes over the 2010–2011 year described the number of processes completed, within categories of 0–3, 4–6, or 7–9. NDA processes include measurements for HbA1c, blood pressure, total cholesterol, serum creatinine, urine albumin-to-creatinine ratio, body mass index (BMI), smoking history, retinal screening, and a foot examination.

### Primary and secondary outcomes

The primary and secondary outcomes were time (from April 1, 2011)-to-first CPRD or HES record of incident non-traumatic minor or major and major-only, respectively, lower limb amputation. The online supplemental material contains CPRD ‘medcode’ and OPCS Classification of Interventions and Procedures version 4 code lists used for the outcome definitions.

### Covariates

The study covariates (measured at baseline) included sociodemographic variables (age, sex, ethnicity, 2010 patient-level Index of Multiple Deprivation (IMD)), geographical region of the individual’s primary care practice, and disease-related variables (time since diagnosis, number of diabetes complications, number of glucose lowering therapies (GLTs) prescribed, and presence of insulin prescription, the latter two measurements made within 6 months prior to baseline). Lifestyle variables (BMI, smoking status, alcohol use) and comorbidities (number of QOF registers on which the individual appeared in 2010–2011, number of hospital admissions in the same year, and number of prescriptions in the 6 months prior to cohort entry) were also included. All covariates were included in the study protocol, which was peer reviewed and approved by the Independent Scientific Advisory Committee for CPRD. Full variable definitions are detailed in the online supplemental material.

### Statistical analysis

Baseline cohort characteristics were described, and extents and patterns of variable missingness were explored. Missing patient-level IMD values were imputed using practice-level IMD data. Missing ethnicity and lifestyle data were imputed from the remaining covariates, using the *mice* package in RStudio V.3.5.1,^18^ with five imputations used. Nearest neighbour propensity score matching was performed using the *matchit* package,^19^ with a caliper of 0.2 for each exposure definition.^20^ Univariate and multivariate Cox proportional hazards models were fitted using the matched samples for each of the exposure definitions with the corresponding exposure as an additional covariate. Concordance statistics were calculated for each of the multivariate Cox proportional hazards models. Sensitivity analyses were performed on both outcomes to assess the effect of QOF indicator attainment among: (1) participants who had met the other two indicators; and (2) those who had not met either of the other two indicators.

## Results

### Summary of cohort characteristics

A total of 83 688 adults (44.3% female) diagnosed with type 2 diabetes before April 1, 2010 and registered across 330 practices were identified as eligible for inclusion. Their baseline characteristics are summarized in table 1. Mean (SD) age was 68.0 (SD 12.5) years, 83.5% were of white ethnic background, and the mean interval since diabetes diagnosis was 7.4 (SD 5.5) years. Most were current or ex-smokers (51.8%), consumed alcohol (70.6%), and/or were overweight or obese (83.4%). Participants had a mean of 2.4 (SD 1.7) comorbidities and 1.7 (SD 1.3) diabetes complications at baseline; and 8.0 (SD 8.8) different prescriptions and 1.3 (SD 1.0) different GLTs in the 6 months preceding study entry. Insulin was prescribed to 11 699 participants (14.0%) during that time.

**Table 1:** Baseline cohort characteristics (N=83 688)

Variable		n or mean	% or SD
Age		67.99	12.49
Sex: female		37 094	44.32
Ethnicity	Asian	4893	5.85
	Black	1754	2.10
	Mixed	555	0.66
	Other	963	1.15
	White	69 880	83.50
	Missing	5643	6.74
IMD quintile	0 (least deprived)	15 998	19.12
	1	18 903	22.59
	2	17 223	20.58
	3	17 097	20.43
	4 (most deprived)	14 424	17.24
	Missing	43	0.05
Region	North East	2162	2.58
	North West	14 654	17.51
	Yorkshire & The Humber	3251	3.88
	East Midlands	1825	2.18
	West Midlands	9959	11.90
	East of England	8651	10.34
	South West	11 352	13.56
	South Central	10 066	12.03
	London	10 959	13.10
	South East Coast	10 809	12.92
Weight status	Underweight	635	0.76
	Ideal weight	12 329	14.73
	Overweight	27 971	33.42
	Obese	41 855	50.01
	Missing	898	1.07
Smoking status	Never smoker	40 161	47.99
	Ex-smoker	31 691	37.87
	Current smoker	11 631	13.90
	Missing	205	0.24
Alcohol (units/week)	0	13 859	16.56
	1–14	49 143	58.72
	15–42	8123	9.71
	>42	1783	2.13
	Missing	10 780	12.88
Number of comorbidities		2.36	1.65
Number of hospitalizations during 2010–2011		0.17	0.54
Duration of diabetes (years)		7.40	5.51
Number of diabetes complications		1.66	1.26
Number of GLT prescriptions within preceding 6 months		1.32	0.99
Insulin prescription within preceding 6 months		11 699	13.98
Number of prescriptions within preceding 6 months		8.02	8.76

GLT, glucose lowering therapy; IMD, Index of Multiple Deprivation.

Over a follow-up of 6.75 years (mean 3.9 (SD 2.0) years), 521 (0.6%) non-traumatic minor or major lower limb amputations were observed, with 309 major-only amputations occurring during this period, corresponding to overall and major-only amputation rates of 1.62 and 0.96 per 1000 person-years, respectively. The observed distribution of QOF indicator attainment and NDA process completion, clustered by the number of indicators/processes achieved, is outlined in table 2. The HbA1c, blood pressure, and cholesterol QOF indicators were met by 54 595 (65.2%), 48 675 (58.2%), and 63 038 (75.3%), respectively, with 27 653 (33.0%) meeting all three indicators. NDA process completion ranged from 54 818 (65.5%; retinal screening) to 80 392 (96.1%; blood pressure measurement). Most (70 673, 84.5%) completed seven to nine NDA processes, but fewer than half (35 462, 42.4%) completed all nine. Those who did not have a measurement during the year of interest were classified as not attaining the corresponding indicator. This included 4759 (5.7%), 3296 (3.9%), and 7680 (9.2%) for HbA1c, blood pressure, and cholesterol, respectively.

**Table 2:** Number (%) of individuals who met each of the QOF indicators and NDA care processes (columns) clustered by the number of QOF indicators or NDA processes met (rows)

Number of indicators/care processes met		Total	QOF indicator met			NDA process completed								
			HbA1c	Blood pressure	Cholesterol	HbA1c	Blood pressure	Cholesterol	Serum creatinine	Urine ACR	Foot examination	BMI	Smoking history	Retinal screening
QOF indicators	0	5742 (6.86%)	0 (0%)	0 (0%)	0 (0%)	3071 (53.48%)	3921 (68.29%)	2559 (44.57%)	3241 (56.44%)	2373 (41.33%)	2850 (49.63%)	3327 (57.94%)	3250 (56.6%)	2981 (51.92%)
	1	17 237 (20.6%)	4825 (27.99%)	4182 (24.26%)	8230 (47.75%)	15 589 (90.44%)	16 461 (95.5%)	14 276 (82.82%)	15 512 (89.99%)	12 260 (71.13%)	13 396 (77.72%)	14 963 (86.81%)	13 809 (80.11%)	10 760 (62.42%)
	2	33 056 (39.5%)	22 117 (66.91%)	16 840 (50.94%)	27 155 (82.15%)	32 616 (98.67%)	32 357 (97.89%)	31 520 (95.35%)	32 281 (97.66%)	26 667 (80.67%)	28 429 (86%)	30 563 (92.46%)	28 239 (85.43%)	22 148 (67%)
	3	27 653 (33.04%)	27 653 (100%)	27 653 (100%)	27 653 (100%)	27 653 (100%)	27 653 (100%)	27 653 (100%)	27 434 (99.21%)	23 703 (85.72%)	24 848 (89.86%)	26 273 (95.01%)	24 387 (88.19%)	18 929 (68.45%)
NDA processes	0–3	3473 (4.15%)	413 (11.89%)	795 (22.89%)	239 (6.88%)	660 (19%)	1581 (45.52%)	343 (9.88%)	732 (21.08%)	160 (4.61%)	294 (8.47%)	460 (13.25%)	627 (18.05%)	1130 (32.54%)
	4–6	9542 (11.4%)	5179 (54.28%)	4929 (51.66%)	5032 (52.74%)	7924 (83.04%)	8547 (89.57%)	6515 (68.28%)	7634 (80%)	2923 (30.63%)	3261 (34.18%)	5467 (57.29%)	4231 (44.34%)	4012 (42.05%)
	7–9	70 673 (84.45%)	49 003 (69.34%)	42 951 (60.77%)	57 767 (81.74%)	70 345 (99.54%)	70 264 (99.42%)	69 150 (97.85%)	70 102 (99.19%)	61 920 (87.61%)	65 968 (93.34%)	69 199 (97.91%)	64 827 (91.73%)	49 676 (70.29%)
	9	35 462 (42.37%)	25 356 (71.5%)	21 820 (61.53%)	30 211 (85.19%)	35 462 (100%)	35 462 (100%)	35 462 (100%)	35 462 (100%)	35 462 (100%)	35 462 (100%)	35 462 (100%)	35 462 (100%)	35 462 (100%)
Total		83 688 (100%)	54 595 (65.24%)	48 675 (58.16%)	63 038 (75.33%)	78 929 (94.31%)	80 392 (96.06%)	76 008 (90.82%)	78 468 (93.76%)	65 003 (77.67%)	69 523 (83.07%)	75 126 (89.77%)	69 685 (83.27%)	54 818 (65.5%)

QOF targets: HbA1c ≤59 mmol/mol (7.5%); blood pressure ≤140/80 mm Hg; total cholesterol ≤5 mmol/L.

ACR, albumin-to-creatinine ratio; BMI, body mass index; HbA1c, glycated hemoglobin; NDA, National Diabetes Audit; QOF, Quality and Outcomes Framework.

### Associations between QOF indicator exposures and lower limb amputation

**Figure 1:**
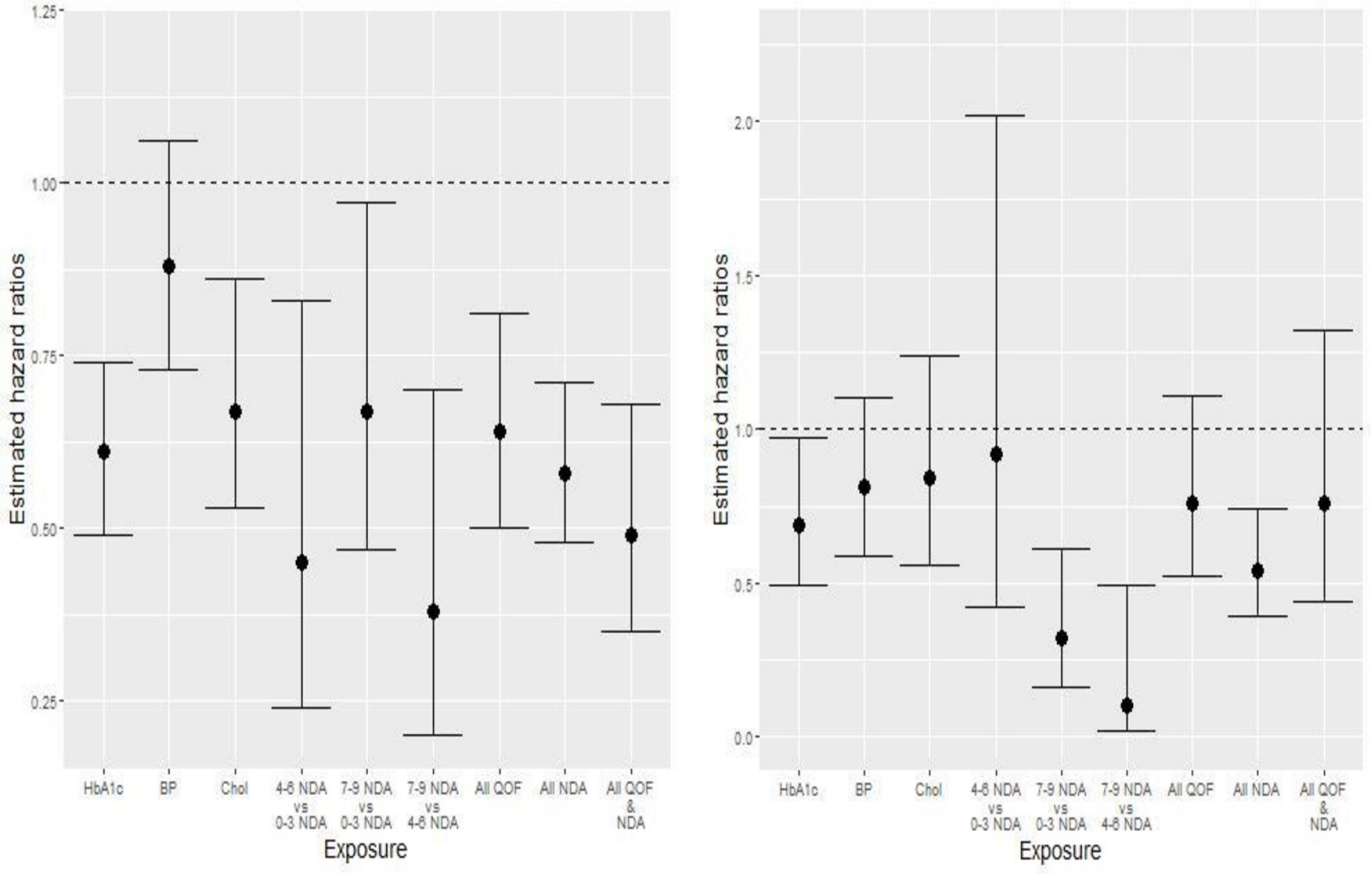
Key HR estimates (and corresponding 95% CIs) for risk of first major or minor amputation (left), and first major amputation (right), across exposure definitions. BP, blood pressure; Chol, cholesterol; Hb1c, glycated hemoglobin; NDA, National Diabetes Audit; QOF, Quality and Outcomes Framework.

The unadjusted and adjusted associations between exposure to attainment of each of the QOF indicators and incident lower limb amputation are displayed in table 3. HbA1c and cholesterol indicator attainment were associated with lower rates of minor or major amputation in both the unadjusted and adjusted analyses (adjusted HRs (95% CI) 0.61 (0.49 to 0.74; p<0.0001) and 0.67 (95% CI 0.53 to 0.86; p=0.0017), respectively). Whereas, attainment of only the QOF HbA1c indicator was significantly associated with lower risk of major-only amputation in both unadjusted and adjusted analyses (adjusted HR 0.69 (95% CI 0.49 to 0.97; p=0.0302)). Blood pressure indicator attainment was not significantly associated with minor or major amputation rates (adjusted HR 0.88 (95% CI 0.73 to 1.06; p=0.19)) or major-only amputation rates when assessing our secondary outcome (adjusted HR 0.81 (95% CI 0.59 to 1.10; p=0.1754)). Though, attainment of all QOF indicators was associated with a 36% decreased risk of major or minor amputation (adjusted HR 0.64 (95% CI 0.5 to 0.81; p=0.0002)). Figure 1 displays key adjusted HR estimates and corresponding 95% CIs across exposures for the primary and secondary outcomes. Full model results (ie, including unadjusted and adjusted HR estimates for all covariates across both outcomes) are available in the online supplemental material.

**Table 3:** Unadjusted and adjusted HRs (with corresponding 95% CIs and p values) for lower limb amputation given QOF indicator exposures/NDA care processes after one-to-one propensity score matching; incidence rates per 1000 person-years are also included for the matched samples

Outcome	Exposure	Incidence rate		Unadjusted analyses			Adjusted analyses*		
		Exposed	Unexposed	HR	95% CI	P value	HR	95% CI	P value
First minor or major amputation	HbA1c QOF target	1.39	2.44	0.57	0.46 to 0.69	<0.0001	0.61	0.49 to 0.74	<0.0001
	Blood pressure QOF target	1.55	1.70	0.91	0.76 to 1.10	0.3278	0.88	0.73 to 1.06	0.1891
	Cholesterol QOF target	1.39	1.96	0.71	0.55 to 0.90	0.0048	0.67	0.53 to 0.86	0.0017
	All QOF targets	1.06	1.66	0.64	0.51 to 0.81	0.0002	0.64	0.50 to 0.81	0.0002
	4–6 (vs 0–3) NDA processes	1.45	2.92	0.49	0.27 to 0.87	0.0149	0.45	0.24 to 0.83	0.0106
	7–9 (vs 0–3) NDA processes	1.77	2.27	0.78	0.55 to 1.11	0.1717	0.67	0.47 to 0.97	0.0319
	7–9 (vs 4–6) NDA processes	1.34	3.04	0.44	0.25 to 0.78	0.0049	0.38	0.20 to 0.70	0.0022
	9 (vs <9) NDA processes	1.15	2.05	0.56	0.46 to 0.67	<0.0001	0.58	0.48 to 0.71	<0.0001
First major amputation	HbA1c QOF target	0.56	0.86	0.65	0.47 to 0.91	0.0114	0.69	0.49 to 0.97	0.0302
	Blood pressure QOF target	0.55	0.65	0.84	0.62 to 1.15	0.2777	0.81	0.59 to 1.10	0.1754
	Cholesterol QOF target	0.60	0.70	0.86	0.58 to 1.27	0.4502	0.84	0.56 to 1.24	0.3739
	All QOF targets	0.45	0.60	0.76	0.52 to 1.10	0.1449	0.76	0.52 to 1.11	0.1595
	4–6 (vs 0–3) NDA processes	1.05	1.34	0.78	0.37 to 1.62	0.5046	0.92	0.42 to 2.02	0.8356
	7–9 (vs 0–3) NDA processes	0.35	0.95	0.36	0.19 to 0.69	0.0021	0.32	0.16 to 0.61	0.0006
	7–9 (vs 4–6) NDA processes	0.15	1.26	0.12	0.03 to 0.53	0.0049	0.10	0.02 to 0.49	0.0042
	9 (vs <9) NDA processes	0.43	0.80	0.52	0.38 to 0.71	<0.0001	0.54	0.39 to 0.74	0.0001

*Adjusted for age, sex, ethnicity, IMD, practice region, BMI, smoking status, alcohol consumption, number of other comorbid conditions, hospitalizations, duration of diabetes, diabetes complications, number of glucose lowering therapies, and insulin prescription status.

BMI, body mass index; HbA1c, glycated hemoglobin; IMD, Index of Multiple Deprivation; NDA, National Diabetes Audit; QOF, Quality and Outcomes Framework.

### Associations between NDA process completion exposures and lower limb amputation

Table 3 presents associations between NDA care process categories and incident lower limb amputation. For all comparisons, substantially lower minor or major amputation rates were observed among those who completed a greater number of care processes after adjustment for potential confounders (adjusted HRs 0.45 (95% CI 0.24 to 0.83; p=0.0106), 0.67 (95% CI 0.47 to 0.97; p=0.0319), and 0.38 (95% CI 0.20 to 0.70; p=0.0022) for comparisons of 4–6 vs 0–3, 7–9 vs 0–3, and 7–9 vs 4–6 completed processes, respectively). Results were relatively consistent for major-only amputation with exception of the 4–6 vs 0–3 comparison. Adjusted HRs are again shown graphically in figure 1 for both outcomes. Additionally, online supplemental figure 1 displays Kaplan-Meier amputation free survival curves and corresponding 95% CIs for each exposure.

Full model results are available in the online supplemental material. Adjusted HRs show the number of complications to be the only covariate consistently significant across all QOF and NDA exposures for either the primary or secondary outcome, which is associated with increased amputation risks ranging from 142% (adjusted HR 2.42 (95% CI 2.10 to 2.78; p<0.0001)) to 220% (adjusted HR 3.2 (95% CI 2.56 to 4.01; p<0.0001)) as well as from 135% (adjusted HR 2.35 (95% CI 2.01 to 2.75; p<0.0001)) to 262% (adjusted HR 3.62 (95% CI 2.62 to 5.01; p<0.0001)) corresponding to the primary and secondary outcomes, respectively. Other covariates (eg, Asian ethnicity) show associations, among various exposures, with significant risk reductions in amputation—whether major or minor, or major-only. Model concordance (C-) statistics range between 0.8409 (95% CI 0.8405 to 0.8413) and 0.9260 (95% CI 0.9255 to 0.9264) as well as between 0.8453 (95% CI 0.8435 to 0.8471) and 0.9499 (95% CI 0.9488 to 0.9510) for the primary and secondary outcomes, respectively, across all exposures, reflecting strong model fit.

### Sensitivity analyses

In the analyses of the QOF indicators restricted to those participants who had met the other two QOF indicators, results for HbA1c and cholesterol indicator attainment were inconsistent for the primary outcome (adjusted HRs 0.83 (95% CI 0.61 to 1.13; p=0.2399) and 1.04 (95% CI 0.59 to 1.84; p=0.8816), respectively), as well as for the secondary outcome for HbA1c (adjusted HR 1.16 (95% CI 0.70 to 1.92; p=0.5575)). Results for blood pressure attainment were consistent (adjusted HRs 0.92 (95% CI 0.65 to 1.29; p=0.6240) and 0.89 (95% CI 0.51 to 1.54; p=0.6823)) for major or minor and major-only amputations, respectively (see online supplemental material). In the analyses restricted to those who had not met either of the other two QOF indicators, results were consistent with the primary analysis for all indicators (adjusted HRs 0.42 (95% CI 0.22 to 0.78; p=0.0064) and 0.19 (95% CI 0.05 to 0.73; p=0.0154) for HbA1c, 0.79 (95% CI 0.53 to 1.17; p=0.2336) and 0.85 (95% CI 0.41 to 1.75; p=0.6615) for blood pressure, and 0.65 (95% CI 0.44 to 0.96; p=0.0316) and 0.85 (95% CI 0.47 to 1.54; p=0.5902) for cholesterol corresponding to the primary and secondary outcomes, respectively) (see online supplemental material).

## Discussion

We investigated the associations between attainment of QOF clinical indicators, completion of NDA care processes, and non-traumatic lower limb amputations among those with type 2 diabetes. We observed that minor or major, as well as major-only, amputation rates were 26%–51% and 3%–51%, respectively, lower among those who met the HbA1c indicator and 14%–47% lower among those who met the cholesterol indicator for our primary outcome. However, we did not find evidence of association between blood pressure indicator attainment and amputation. Sensitivity analyses for both outcomes were indicative that the incremental gains associated with HbA1c and cholesterol indicator attainment may be lower when greater numbers of QOF indicators have been met overall, in keeping with the idea that relative risk reduction is greater where baseline absolute risk is higher. Substantially lower amputation rates across both outcomes were also observed among those who completed a greater number of NDA care processes, indicative of benefits of comprehensive care. Although formal trial evidence regarding the association between HbA1c control and amputations is relatively sparse in view of amputations being a relatively infrequent outcome,^21 22^ our HbA1c indicator findings correspond with results of a number of observational studies.^23–26^ These suggest a relatively linear relationship between HbA1c and amputations, at least within the limits of glycemic control considered optimal in terms of other important diabetes complications.^23 24 26^ Previous observational and randomized studies are similarly in agreement with our findings relating to the cholesterol indicator.^27 28^

Regarding blood pressure control, although this plays an essential role in the management of type 2 diabetes as part of comprehensive secondary prevention, after adjustment for other study covariates, we did not find an association between blood pressure (at the QOF indicator threshold) and lower limb amputation. This corresponds with the findings of some,^29^ but not all,^30^ previous studies. Residual confounding may be relevant to the apparent lack of association. Hypertension is a well-established risk factor for peripheral arterial disease.^31^ However, the interplay between peripheral arterial disease, blood pressure, and foot perfusion is not clearly understood, and it may be the case that relatively low blood pressure is unfavorable in the context of compromised vascular supply (ie, those with existing vascular disease). There may also be an element of reverse causation, as autonomic neuropathy can lead to relative hypotension, and neuropathy is part of the pathological process that results in amputation among those with diabetes.^32^

Overall, our findings generally support use of the current QOF and NDA indicators in reducing the risk of amputations. The reduction in amputation rates seen over the last decade^4^ may be due to the emphasis placed on secondary prevention through QOF over the last 17 years, given the associations between QOF indicator achievement, care process delivery, and amputation incidence that we demonstrate in our study. More refined analyses among those in whom relative hypotension does not reflect underlying disease would aid interpretation of the blood pressure findings. And indeed, the QOF blood pressure indicator was amended in 2019–2020, such that it no longer applies to those with moderate-severe frailty, who are at potentially greatest risk of blood pressure effects of comorbidities.^33^ Regarding the potentially more limited benefit of meeting QOF indicators where relatively comprehensive care is already achieved, this makes theoretical sense and is supported by the trend in point estimates observed in our sensitivity analyses. However, these analyses will also have been relatively underpowered (reflected in the larger CIs), hence we would not conclude that control to indicator levels is not beneficial where other indicators are met. Even if this were the case, we have previously observed that indicator attainment in this context is associated with lower occurrence of mortality and hospital admissions (manuscripts currently under review).

In terms of mechanisms to further type 2 diabetes prevention activities to help mitigate against ongoing increases in lower limb amputation events, it will be important that this is balanced against the disutility associated with comprehensive diabetes care (particularly as this is potentially not dissimilar to that associated with the conditions that predispose to amputation, and amputation is a relatively rare outcome).^3^ Strategies to reduce the disutility associated with diabetes care would be helpful. These should be guided by those with diabetes, but as examples, adaptations could include facilitating safer and less financially costly access to dietary and physical activity options, providing easy-to-negotiate follow-up mechanisms, and inclusive care with strong continuity.

The strengths of this study include the sample size and that the sample is likely to be reasonably representative of the population of interest. The CPRD employs routine quality assurance checks,^34 35^ and CPRD data have been shown to offer clinical predictive value and similar incidence to other sources of UK health data.^34 35^ They have previously been successfully used for validation of amputation risk predictive modelling among those with type 2 diabetes,^24^ and the dataset has a linkage to non-primary care data including hospital admissions on a prospective basis. The dataset enabled adjustment for many potential confounders. QOF incentivizes use of standard recording methods, and HES data are widely used and considered to be of high quality. HES data are subject to regular national audits, and a systematic review has evaluated its accuracy as high for both diagnoses and procedures.^36^

There are, nonetheless, important limitations to our study. An element of miscoding and misclassification will have occurred as a result of inconsistency in general practitioner coding practices. That we defined our exposure using 2010–2011 data, and did not assess variation over time, will also have led to some heterogeneity in clinical status as related to exposure classification and potential dilution of effect. For the major-only secondary outcome, some individuals had prior minor amputations during the study period. Our study population excludes those with amputation history at baseline, who may experience different incidence rates. Additionally, the events and rates reported correspond to first amputations (whether overall (primary analysis) or major-only (secondary analysis)), where censoring occurs at the time of first amputation, rather than overall amputation rates during the study period for this cohort. It is also possible that the exposures could, to an extent, reflect level of engagement with care more generally, as much as their specific physical correlates. Our interpretation of the analyses of NDA care processes is limited by their examination as a ‘count’ measure, rather than individual processes. It would be anticipated that some processes, such as foot examination, would be particularly relevant to amputation prevention.^37–39^ Finally, mortality-driven censoring could be more prevalent among those with higher amputation risks,^40^ with different distributions between exposed and unexposed groups. Thus, the Cox proportional hazards assumption of non-informative censoring may not apply to all individuals.

Both comprehensive diabetes care, and HbA1c and cholesterol control to the levels investigated, are associated with considerably lower risks of lower limb amputation. This information may help inform individual diabetes management and medical decision-making, as well as diabetes-related resource allocation. It supports widening access to comprehensive diabetes care. Inclusive discussions around reducing care disutility may help promote both equity in access and care uptake.

## Data Availability

Data may be obtained from a third party and are not publicly available. We are unable to make data available because of CPRD license restrictions.
