# Supplementary material for: Associations between attainment of incentivized primary care indicators and incident lower limb amputation among those with type 2 diabetes: a population-based historical cohort study
Source: BMJ Open Diabetes Res Care. 2021 Apr 26;9(1):e002069. doi: 10.1136/bmjdrc-2020-002069 (PMC8076942; doi:10.1136/bmjdrc-2020-002069)

Online Supplemental Material

Code lists used in cohort derivation and variable definitions

Codes listed are CPRD medcodes or prodcodes unless stated otherwise.

Type 2 diabetes mellitus

108018 108005 107701 107508 107331 106528 106061 105784 104639 104323 103902 103543 102611 102201 101801 100964 98723 98616 95351 95159 95093 93727 93657 93530 93529 91646 85991 83532 72320 70316

69278 67905 66965 65704 65267 64668 64571 63690 63357 62674 62146 62107 61071 60796 60699 59725 59365 59253 58604 57278 56803 56268 55842 55075 54899 54212 53392 52303 51756 50813

50527 50429 50225 49869 49655 49074 48192 47954 47816 47409 47321 47315 46917 46150 45919 45913 45467 44982 44779 43785 43227 43139 42762 41389 40962 40401 39317 37806 37648 36633

35385 35105 34912 34450 34268 33807 32627 29979 26054 25627 25591 24836 24693 24458 22884 18777 18496 18425 18390 18278 18264 18219 18209 18143 17859 17262 14803 12736 12640 11047

8403 5884 4513 1684 1407 758 506

Type 1 diabetes mellitus

108724 108360 108007 105337 104453 102946 102740 102704 102620 102163 102112 101735 101311 100770 99719 99716 99311 99231 98704 98071 97894 97474 97446 96235 95992 95343 93922 93878 93875 93468

91943 91942 85660 72702 70766 70448 69993 69748 69676 69124 68792 68390 68105 67853 66872 66145 65616 64446 63017 62613 62352 62209 61829 61344 60499 60208 60107 57621 56448 55239

54600 54008 53200 52283 52104 51957 51261 49949 49554 49276 49146 47650 47649 47582 46963 46850 46301 45914 45276 44443 44440 44260 43921 42831 42729 42567 41716 41049 40837 40682

40023 39809 39070 38161 35288 30323 30294 26855 24694 24490 24423 22871 21983 18683 18642 18505 18387 18230 17858 17545 12455 10692 10418 6791 6509 1647 1549 1038

Other (i.e. not type 1 or type 2) specified diabetes diagnosis

100347 98392 96506 95636 95539 94383 93380 67212 66675 64283 61523 61122 59991 59288 52236 51697 47377 46624 46290 38617 33969 31310 26108 23479 22487 14889 13279 11551 10098

Ethnic background- White

101787 101219 100143 99808 99788 98213 98111 55223 55113 47949 47601 47074 46956 45955 45947 42294 42290 40102 32778 32413 32126 32069 32066 28973 28936 28900 28887 28866 26391 26341 26310 25422 24270 22467 12769 12746 12681 12591 12532 12467 12444 12436 12433 12421 12412 12402 12355 12352 12351

Ethnic background- Asian

12414 12460 12482 12513 12608 12668 12760 12887 24690 24740 25920 26379 26392 28888 28935 32396 47077 64133

Ethnic background- Black

93144 57763 57753 57752 57435 57094 57075 54593 50286 48005 47965 46812 46047 41329 40097 35412 35350 32886 32389 32136 26312 24339 12778 12632 12452 12443 12432 12350

Ethnic background- Mixed

49940 47401 47005 46056 40110 40096 35459 32443 32425 32420 32408 32401 32399 32165 25676 25623 12873 12795 12742 12706 12696 12653 12638 12437

Ethnic background- Other

101162 99316 96789 94487 89910 85505 71425 64610 64609 63872 60837 57764 57286 56127 55584 49658 47969 47285 47091 47028 46964 46818 46752 46649 46063 46059 45964 45199 45008 41214 39696 38097 32781 32479 32382 32110 30280 28909 26455 26246 25937 25451 25434 25411 25082 24962 24272 12757 12756 12730 12719 12718 12633 12473 12468 12434 12420

Nephropathy

109805 109980 110626 109905 109904 109990 109657 109963 109804 95145 95188 95571 95176 100633 95180 97587 99312 104619 104963 95406 95122 95177 95178 95175 95408 95123 94793 95179 94965 12479

12566 12585 95508 99160 95405 97683 110133 109981 110467 105328 109455 53852 6712 105151 8330 104905 104960 107000 104630 104201 106620 105811 108437 106866 49028 70874 5504 94964 31549 48022

64636 56760 36442 59194 83513 65089 17253 48057 100693 70712 54990 18774 28158 66714 5911 22252 46145 20196 44422 60302 2997 11745 66705 24361 98364 89924 96133 72004 11773 20073

2994 2996 88597 30756 64828 8037 11553 85991 10924 18390 110484 110108 110251 110003 13590 13611 13621 13600 9430 28180 5451 28180 1802

Neuropathy

109197 106360 104323 103966 103902 102859 102434 102274 99855 98616 96256 95351 94821 91646 81439 72922 72889 72320 66965 63690 63555 62674 62384 59903 59253 57333 50813 50527 50099 49640

49575 49074 48078 47816 47409 45467 44113 44033 40962 39317 37759 37315 36643 35785 35537 35385 35316 35116 34268 31790 31097 29881 27891 24571 24331 24226 22573 21781 18492 18425

18142 17247 17067 16540 16491 16452 16293 16230 12790 12736 12066 11663 11544 9881 8710 7795 5002 3958 3838 2790 2342 710

Retinopathy

111798 104263 101881 100979 86068 65463 58604 57382 52630 52041 49655 47584 47328 46068 45876 43377 42762 41229 40982 39457 38096 36867 36119 36035 35659 34455 30648 30477 25888 25591

22967 19533 18775 18662 18496 17262 13108 13107 13103 13102 13101 13099 13097 11626 11599 11433 11129 11018 10882 10755 10701 10099 9835 9339 9318 8237 7890 7069 6702 3837

3286 3285 2986 2653 1438 1323

Ischemic heart disease

107406 18249 8312 9414 7134 31556 32651 19402 36011 33461 37682 28837 96804 2901 5703 24888 8679 7634 7442 11610 44561 19413 10209 42708 70111 57241 45886 45370 92419 66664

66236 67761 52938 67554 31540 101569 63153 33718 48822 92233 31519 44723 51507 22647 68123 68139 62608 67591 60753 5744 18670 33735 42462 86071 22828 33650 40996 66583 19046 8942

42304 93618 35674 40399 6336 50372 57062 45476 100139 105216 103655 13185 19542 15373 14782 29300 108504 108506 15349 7137 51515 61310 7609 59423 48767 19193 97953 57634 37719 56990

72780 41547 732 6182 31679 33620 22020 69247 44585 55598 55092 93828 70755 34963 3159 33471 105184 31571 10603 43939 60067 87849 85947 92927 96537 61208 45960 101121 101373 240

24783 20416 1792 241 13566 2491 30421 1204 1677 13571 17689 12139 5387 40429 17872 14897 8935 29643 23892 14898 63467 3704 9507 10562 1678 30330 17133 32854 29758 12229

34803 28736 62626 41221 46017 14658 27951 23579 36523 4656 1431 19655 61072 55137 7347 17307 34328 18118 11983 54251 9276 68357 27977 4017 16408 17464

1430 25842 66388 54535 7696 1414 9555 26863 12804 28554 5413 1655 1344 3999 5254 6331 27484 2155 67087 105250 41677 36609 7320 34633

24540 23078 35713 18842 45809 38609 72562 46166 36423 24126 23708 37657 59189 59940 69474 29553 32272 46112 46276 106812 41835 68748 22383 1676 52517 39546

68401 47637 96838 109035 99991 69776

Ischemic cerebrovascular disease

110337 107886 105738 105202 105100 104517 104505 101670 101251 98642 97003 96744 95347 94482 93770 93459 93134 92036 91775 91627 90572 89365 73022 71585 70235 68906 68905 68366 68329 68069

66873 66489 63746 62661 57909 57495 55351 55074 53818 53745 52008 51767 51326 50594 50242 47642 47580 45781 44765 44023 43449 41703 40847 40758 40347 39780 39403 36717 35916 34758

34135 33543 33499 33377 32447 29973 27975 26424 25910 25615 24446 23942 23671 21669 21118 20811 20672 19354 19348 19280 19260 18689 18686 17322 16956 16517 15788 15252 15019 15007

13567 12833 12733 11928 10792 10504 9985 8837 8443 8149 7780 7720 7138 6305 6253 6228 6155 6116 5871 5602 5363 5185 4240 4152 3602 3149 2654 1895 1469 1433

1298 1195 569 504

Peripheral vascular disease

112218 111593 111190 111037 108792 108721 108267 107740 107652 107158 106020 105773 105730 105317 104364 101910 101866 100835 100814 100113 100036 99676 99532 98801 98574 98174 97895 97606 97164 96809

96255 81445 73822 72491 72448 71860 71110 70922 69519 69232 68412 68320 68141 67982 67818 67083 66930 66917 66879 66869 66820 66804 66437 65692 65669 65286 64555 63711 63605 63589

63396 63368 63280 63238 62866 62818 62775 62099 61974 61256 61255 60867 60693 60465 60212 59602 59535 59187 57822 57793 56919 55877 55554 55324 54865 54211 53675 53580 52869 52695

52473 52462 52357 52342 52289 52033 51720 51331 51330 51211 51017 50894 50589 50580 49560 49319 49273 48939 48929 48899 48846 48700 48492 47940 47889 47835 47562 47538 46465 46186

46168 45428 44835 44430 44250 44097 43651 43648 42645 42640 42115 41823 41768 41583 40732 40397 39877 39776 39553 39437 39436 39039 38921 37958 37787 36443 36136 36065 34037 33714

33621 33565 33555 32634 32492 32049 31916 31785 31338 30989 29112 28894 28777 28651 28616 28130 28125 28119 28030 27580 27494 27350 27320 26213 25347 25248 24692 24677 24667 24229

24221 24097 22016 21927 21290 21167 20892 18816 18778 18062 18060 18038 18030 17336 16363 16207 15915 15170 15071 15010 12563 12331 12206 11766 11765 11242 10827 9554 9119 9099

8610 7694 7111 6853 6617 6356 6256 4539 3779 3701 3590 3530 3344 3245 2760 2361 2066 2065 1517

Acarbose

9105 5621 5174 479

DPP-4 inhibitor

60682 60681 60497 60328 59809 59385 59177 58865 56965 54973 54891 54150 50682 50124 50087 48533 48401 46716 46665 45821 45775 43684 43619 41431 41204 39203 39149 38551 37902 37875

37874 35462 35022

SGLT2-inhibitor

60643 60430 60386 60379 60211 60073 60066 60012 54480 54265 54203 54182

GLP1 agonist

55767 55729 55728 55723 55459 55413 46469 46458 40693 40642 35251 35150 35149 35144

Insulin

61562 60967 60951 60938 60933 60750 60626 60621 60609 60028 59846 59793 59533 59500 59475 59311 59243 59133 59005 59004 58995 58961 58884 58878 58817 58801 58798 58754 58745 58581

58579 58578 58449 57744 57622 57620 57564 57529 57493 57451 57388 57387 57243 57153 56983 56939 56879 56857 56808 56785 56691 56656 56642 56639 56624 56502 56495 56489 56352 56115

55910 55907 55746 55687 55627 55618 55603 55521 55517 55462 55234 54886 54885 54629 54573 54462 54028 54027 53710 53437 53251 53148 53118 52748 52722 52522 52319 52232 51881 51836

51743 51650 51612 51182 51107 50798 50691 50633 49831 49509 49508 49507 49506 49479 49451 49307 49172 49108 49052 48829 48811 48771 48765 48633 48576 48501 48435 48342 47856 47751

47588 47360 46666 46001 45639 45158 45045 44810 44601 44480 44378 44251 43991 43953 43950 43833 43670 43568 43489 42954 42797 42395 42305 41959 41834 41120 40555 40085 39150 39086

39006 38986 38808 38774 38422 38236 38093 37427 37055 36959 36920 36853 36513 36430 36356 36355 36194 36146 36066 36043 36031 35701 35468 35454 35260 35253 35218 35143 35081 35078

35057 35017 34713 34097 34031 33966 33914 33356 33232 33167 33101 32053 31699 31467 31466 31465 31464 31267 31258 31205 30918 30861 30819 30686 30305 30236 30209 29953 29837 29567

29090 28978 28723 28666 28588 28442 28185 28183 28101 28096 27911 27614 27461 27402 27396 27280 27177 27151 27149 26795 26784 26621 26498 26403 26338 26098 26060 25812 25786 25736

25735 25479 25422 25133 25006 24993 24866 24846 24845 24800 24795 24722 24593 24554 24485 24002 23993 23992 23636 23437 23231 23099 23003 22987 22983 22974 22946 22945 22823 22806

22697 22496 22328 22161 22155 22094 22060 22058 21945 21590 21583 21554 21459 21422 21395 21374 21347 21235 21232 21223 21110 20995 20672 20671 20422 20196 20195 19977 19878 19877

19829 19707 19513 19491 19271 19029 18931 18645 18593 18592 18590 18461 18446 18301 18224 18208 18195 18149 17809 17731 17712 17643 17405 17377 17336 17076 16959 16866 16700 16682

16389 16209 16160 16152 16142 16129 15961 15951 15895 15710 15624 15484 15294 15199 15040 14944 14938 14933 14930 14928 14925 14918 14887 14649 14646 14644 14642 14619 14506 14505

14504 14362 14357 14345 14340 14339 14330 14313 14301 14299 14290 14270 14191 13969 13837 13819 13729 13622 13550 13516 13474 13416 13331 13277 13274 13108 13096 13036 13009 12892

12840 12818 12654 12638 12455 12300 12299 12297 12244 12060 12035 11878 11521 11408 11346 11345 11337 11271 11245 11107 11086 11080 11056 11055 10915 10910 10887 10691 10572 10566

10547 10546 10545 10484 10277 10264 10259 10258 10245 10244 10243 10242 10229 10225 10208 10207 10184 10175 10145 10133 10067 10001 9834 9737 9702 9619 9618 9578 9565 9521

9503 9376 9363 9341 9108 9079 8895 8841 8839 8838 8646 8483 8376 8354 8322 8203 8118 7959 7861 7793 7783 7772 7771 7765 7764 7763 7757 7537 7412 7402

7400 7393 7350 7349 7319 7318 7300 7267 7266 7237 7231 7228 7203 7164 7127 7075 7062 6991 6981 6965 6958 6831 6781 6753 6730 6724 6554 6470 6447 6378

6233 6228 6209 6138 6091 6061 6060 6057 6009 5967 5966 5962 5953 5933 5892 5891 5873 5850 5845 5789 5769 5742 5649 5634 5620 5557 5501 5421 5345 5267

5255 5250 5214 5164 5142 5121 5059 5022 5021 5015 4896 4790 4784 4760 4715 4706 4248 4247 4199 4198 4163 4129 4093 3551 3550 3439 3396 3076 2929 2812

2808 2459 2456 2455 2454 2373 2321 2221 2220 1886 1844 1843 1842 1840 1839 1806 1805 1751 1649 1645 1643 1595 1594 1593 1592 1591 1589 1588 1587 322

321

Glinide

52203 36948 36774 35561 27125 23945 15955 11483 11366 11321 11316 9865 9748 9707 5989 5678

Metformin

61559 61043 60968 60643 60497 60286 60074 60012 59620 59385 58865 58607 58051 57457 57147 56965 55739 55711 55270 54973 54898 54891 54442 54150 53867 53774 53478 52634 52442 52221

51527 51135 51080 50970 50821 50682 50570 49738 49502 48149 47939 46989 45581 44250 43684 43619 43270 42161 40233 40110 40007 39988 39729 39598 39560 39203 38551 38400 38355 37902

37874 34917 34836 34742 34697 34598 34504 34323 34135 34020 34004 33674 33087 31146 31077 30316 27501 26258 25678 18220 17580 16044 14164 11990 11760 11737 11717 11610 11609 11604

11601 7610 7375 7325 7166 7048 6855 735 93 23

Sulphonylurea

61311 60495 58882 57830 57601 56437 56376 56008 55862 54764 53288 51955 48056 47894 47074 46927 45831 45215 44738 44473 44304 43465 43065 42790 41898 41593 41559 41558 40425 40365

36856 34957 34932 34802 34706 34676 34563 34507 34399 33673 33562 31474 31212 30460 29939 29326 28708 27969 26218 26118 25636 24848 22858 22636 22614 22145 21892 21870 21832 21564

21489 21424 19728 19658 19336 17706 17698 17343 16602 16211 15374 13331 12513 12455 12259 12245 11946 11695 11284 10427 9108 8976 8390 8168 8034 7912 7744 7409 7332 7284

6337 5636 5627 5353 5316 5276 4862 4426 2219 1965 1964 1847 1254 1253 547 32

Thiazolidinedione

57659 56831 56376 56208 48139 48120 37617 31077 30316 20889 20287 19472 18220 17580 15232 14164 13628 11760 11737 11717 11610 11609 11604 11601 10051 9699 9662 7375 7325 6855

5227 548 469

BMI- underweight

32914 29029 26473 24496 12530 126

BMI- reference weight

44291 28946 25061

BMI- overweight

103499 32974 23376 16404 9015 2839

BMI- obese

104421 104129 103574 73304 70950 70898 69757 67517 67516 66406 64712 59780 55586 55585 52735 52036 52034 49409 49250 47439 40153 38799 38658 38632 38294 38059 32843 29538 27570 25968 24755 22695 22556 21744 17477 17444 16196 13278 11401 8854 7984 3176 430

Current smoker

105501 104310 103507 101338 100099 98347 62686 46654 46321 41979 31114 30762 30423 12967 12966 12965 12964 12963 12960 12958 12952 12951 12947 12945 12944 12943 12942 12941 12240 10558 7622 3568 1878 1823 1822 93

Ex-smoker

100963 100495 99838 98447 97210 26470 19488 12961 12959 12957 12956 12955 12946 12878 776 90

Never smoker

33

Alcohol use

CPRD enttype 5, or medcode indicating:

a. No alcohol consumption: 12979 12970 4447
b. 1-14 units/week: 26472 12980 12975 12972 385
c. 15-42 units/week: 12985 322
d. >42 units/week: 104611 103459 102665 102448 102247 101718 100474 97680 97261 96054 96053 94670 94553 73876 69691 68111 67651 65932 65754 64101 62000 59574 57714 56947 56410 54505 48241 47555 47123 43193

   41920 40530 39799 39327 38061 37946 37691 37605 36748 36296 35330 33839 33670 33635 32964 32927 31443 30695 30604 30404 30162 29691 28780 28150 27342 26323 26106 25110 24984 24064

   22277 21879 21713 21650 21624 21412 20762 20514 19494 19401 18636 18156 17607 17330 17259 16225 12984 12982 12977 12976 12974 12554 12496 12442 12353 11670 11106 10691 9849 9489
   8999 8430 8388 8363 8030 7943 7885 7746 7602 7123 6467 6169 5758 5740 4915 4743 4506 4500 3216 2925 2084 2083 2082 2081 1618 1476 1399 669

Variable definitions

1. Exposures

a. Attainment of QOF HbA1c (≤59 mmol/mol; 7.5%) indicator in year 2010-2011, as per QOF Business Rules v38.0

b. Attainment of QOF blood pressure (≤140/80 mmHg) indicator in year 2010-2011, as per QOF Business Rules v38.0

c. Attainment of QOF total cholesterol (≤5 mmol/l) indicator in year 2010-2011, as per QOF Business Rules v38.0

d. Number NDA care processes met in year 2010-2011, as per National Diabetes Audit Primary Care Extraction Specification 2015-16, categorised as 0-3, 4-6, or 7-9 processes

2. Outcomes

a. Non-traumatic minor or major lower limb amputation: CPRD medcodes

6955	15011	32049	69518	12206	25347	12563	21167	11765	11242	105730	111593	3701
27350	47940	50589	52033	111037	18062	3779	100835	71110	2361	97164	62099	18778
39553	100814	104364	50580	3590	97895	54211	108792	51330	15010	3344	15170	31916
27320	107652	59535	48929	33565	49560	28130	51017	37958	47889	16207	60867	3245
25248	48492	33621	26213	33714	31785	48899	15915	15071	98801			

OPCS Classification of Interventions and Procedures (OPCS-4) codes:

X10	X10.1	X10.2	X10.3	X10.4	X10.8	X10.9	X11	X11.1	X11.2	X11.8	X11.9	X09.2
X09.3	X09.4	X09.5	X09.8									

b. Non-traumatic major-only lower limb amputation: CPRD medcodes

6955	15011	32049	69518	12206	25347	12563	21167	11765	11242	3701	27350	47940
50589	52033	111037	18062	3779	100835	71110	2361	97164	62099	18778	39553	100814
104364	50580	3590	97895	54211	108792	51330	15010					

OPCS-4 codes:

X09.2 X09.3 X09.4 X09.5 X09.8

3. Covariates

Sociodemographic variables:

a. Age on 31 March 2011, assuming the birth date to be the earliest possible in the CPRD-recorded birth year

b. Sex as per CPRD record

c. Ethnic background: as per CPRD record where available, and otherwise HES record, collapsed into a categorical variable based on the five Level 1 ONS ethnic group classifications derived for the 2001 Census (see Section 1). The earliest-recorded ethnicity category was used where more than one was available.

d. Index of multiple deprivation: 2010 Index of Multiple Deprivation patient-level score, represented by quintile

e. Region in which the primary care practice is based: as per CPRD record (i.e. North East, North West, Yorkshire and The Humber, East Midlands, West Midlands, East of England, South West, South Central, South East Coast or London)

Lifestyle variables:

f. Body mass index (BMI): the most recent BMI category recorded, or category within which a continuous BMI measurement falls, at baseline – i.e. prior to April 1, 2011

g. Smoking status: categorised as current-, ex- or never- smoker, as per the most recent smoking-related code available, at baseline – i.e. prior to April 1, 2011

h. Alcohol use: weekly consumption of alcohol (units), as per the most recent record (at baseline – i.e. prior to April 1, 2011) indicative of levels of use, categorised as 'no alcohol use', '≤14 units/week, 15-42 units/week, or 43+ units/week

Disease-related variables:

i. Duration of diabetes: time between date of first diabetes-related code in the patient record and March 31, 2011

j. Number of diabetes complications: total number of the following diagnoses recorded in the patient CPRD record prior to April 1, 2011 (i.e. maximum 6): nephropathy, retinopathy, neuropathy, ischemic heart disease, ischemic cerebrovascular disease, peripheral vascular disease

k. Baseline glucose lowering therapy: Number of classes of glucose lowering therapy prescribed in the six months prior to April 1, 2011 (possible classes = metformin, sulphonylureas, insulin, DPP-4 inhibitors, thiazolidinediones, SGLT-2 inhibitors, GLP1 agonists, meglitinides and acarbose, i.e. maximum score = 9)

l. Insulin prescription within the six months prior to April 1, 2011: as per CPRD record

Comorbidity variables:

m. Morbidity burden: total number of QOF registers the patient would have been eligible to appear on in 2010-11, per the QOF Business Rules v 38.0. i.e. maximum 19 from the following list: asthma, atrial fibrillation, hypertension, cancer, chronic obstructive pulmonary disease, coronary heart disease, chronic kidney disease, dementia, depression, epilepsy, heart

failure, learning disability, schizophrenia, bipolar affective disorder or other psychoses, osteoporosis, peripheral arterial disease, palliative care, rheumatoid arthritis, stroke or transient ischemic attack

n. Prescribing burden: the number of unique therapy product code*event date combinations in the patient's CPRD record in the six months prior to April 1, 2011

o. Hospital admissions in 2010-11 year: number of unique HES spells recorded for the individual in the baseline year

Supplementary Table 1: Univariate hazard ratios (with corresponding 95% CIs and p-values) for minor or major amputation risk by each covariate across QOF exposure definitions after 1:1 propensity score matching.

	**Exposure Definition**								
	**Achieve HbA1c QOF Target**			**Achieve Blood Pressure QOF Target**			**Achieve Cholesterol QOF Target**		
	**Hazard Ratio**	**95% CI**	**p**	**Hazard Ratio**	**95% CI**	**p**	**Hazard Ratio**	**95% CI**	**p**
Exposure	0.57	0.46-0.69	<0.0001	0.91	0.76-1.10	0.3278	0.71	0.55-0.90	0.0048
Age	1.02	1.01-1.03	<0.0001	1.01	1.00-1.02	0.0288	1.01	1.00-1.02	0.0764
Sex: Female	0.52	0.42-0.64	<0.0001	0.55	0.45-0.67	<0.0001	0.48	0.37-0.62	<0.0001
Ethnicity: Asian	0.27	0.14-0.52	0.0001	0.37	0.21-0.66	0.0007	0.50	0.26-0.96	0.0388
Ethnicity: Black	0.79	0.41-1.53	0.4793	0.91	0.50-1.66	0.7590	0.64	0.26-1.55	0.3207
Ethnicity: Mixed	0.90	0.29-2.80	0.8555	0.83	0.27-2.57	0.7401	0.48	0.07-3.42	0.4630
Ethnicity: Other	0.36	0.09-1.44	0.1471	0.33	0.08-1.33	0.1182	0.25	0.04-1.79	0.1687
IMD	1.01	0.99-1.02	0.4851	1.01	0.99-1.03	0.2231	1.02	1.00-1.04	0.0630
North West	1.12	0.87-1.43	0.3842	1.15	0.91-1.45	0.2406	1.10	0.81-1.49	0.5452
Yorkshire & Humber	1.06	0.64-1.74	0.8277	0.61	0.34-1.11	0.1063	0.71	0.34-1.51	0.3777
East Midlands	0.78	0.37-1.64	0.5043	1.02	0.55-1.92	0.9393	1.01	0.45-2.27	0.9757
West Midlands	0.70	0.49-0.98	0.0398	0.71	0.51-0.99	0.0422	0.69	0.45-1.05	0.0844
East of England	0.93	0.67-1.30	0.6876	0.95	0.69-1.29	0.7323	1.16	0.80-1.68	0.4444
South West	1.14	0.87-1.50	0.3532	1.27	0.99-1.63	0.0601	1.26	0.91-1.75	0.1556
South Central	1.08	0.81-1.45	0.6057	1.06	0.80-1.40	0.6849	1.02	0.71-1.46	0.9295
London	1.14	0.86-1.50	0.3658	1.14	0.88-1.48	0.3303	1.01	0.71-1.43	0.9624
South East Coast	0.88	0.65-1.20	0.4199	0.83	0.62-1.12	0.2150	0.90	0.62-1.30	0.5686
BMI: Underweight	1.23	0.93-1.63	0.1381	1.17	0.90-1.52	0.2394	1.19	0.86-1.65	0.2936
BMI: Overweight	0.94	0.76-1.17	0.5962	0.92	0.75-1.12	0.4109	1.00	0.78-1.30	0.9729
BMI: Obese	0.94	0.77-1.14	0.5207	0.97	0.81-1.17	0.7653	0.90	0.71-1.15	0.4040
BMI: Missing	1.26	0.56-2.82	0.5742	1.18	0.53-2.64	0.6893	1.23	0.46-3.29	0.6843
Ex-Smoker	1.37	1.12-1.67	0.0020	1.16	0.96-1.40	0.1194	1.32	1.04-1.69	0.0239
Current Smoker	1.36	1.06-1.75	0.0150	1.58	1.26-1.99	0.0001	1.66	1.25-2.20	0.0004
Smoking: Missing	1.44	0.36-5.77	0.6090	1.44	0.36-5.79	0.6056	1.18	0.17-8.43	0.8673
Alcohol: 1-14	0.81	0.67-0.99	0.0401	0.82	0.68-0.99	0.0346	0.83	0.65-1.06	0.1343
Alcohol: 15-42	1.03	0.74-1.44	0.8609	0.91	0.66-1.25	0.5655	0.94	0.63-1.43	0.7847
Alcohol: >42	2.60	1.66-4.08	<0.0001	2.09	1.33-3.27	0.0013	2.87	1.78-4.63	<0.0001
Alcohol: Missing	1.00	0.76-1.33	0.9843	1.06	0.81-1.39	0.6525	1.04	0.74-1.46	0.8157
Morbidities	1.32	1.25-1.39	<0.0001	1.31	1.25-1.38	<0.0001	1.26	1.18-1.35	<0.0001
Prescriptions	1.02	1.01-1.02	<0.0001	1.02	1.02-1.03	<0.0001	1.02	1.01-1.03	<0.0001
Hospitalisations	1.57	1.45-1.69	<0.0001	1.59	1.47-1.72	<0.0001	1.48	1.32-1.66	<0.0001
Duration of diabetes (years)	1.02	1.01-1.04	0.0060	1.03	1.01-1.04	0.0001	1.03	1.01-1.05	0.0018
Complications	2.60	2.42-2.78	<0.0001	2.65	2.49-2.83	<0.0001	2.70	2.49-2.93	<0.0001
Glucose lowering therapies	1.15	1.04-1.27	0.0054	1.31	1.20-1.43	<0.0001	1.35	1.21-1.51	<0.0001
Insulin prescription	3.44	2.82-4.20	<0.0001	4.07	3.37-4.92	<0.0001	3.45	2.68-4.44	<0.0001

Study sizes across exposures after 1:1 propensity score matching are provided in Supplementary Table 4, as they are the same between univariate and multivariate analyses.

Supplementary Table 2: Univariate hazard ratios (with corresponding 95% CIs and p-values) for minor or major amputation risk by each covariate across NDA exposure definitions after 1:1 propensity score matching.

	**Exposure Definition**								
	**Meet 4-6 NDA Processes (vs. Meet 0-3 NDA Processes)**			**Meet 7-9 NDA Processes (vs. Meet 0-3 NDA Processes)**			**Meet 7-9 NDA Processes (vs. Meet 4-6 NDA Processes)**		
	**Hazard Ratio**	**95% CI**	**p**	**Hazard Ratio**	**95% CI**	**p**	**Hazard Ratio**	**95% CI**	**p**
Exposure	0.49	0.27-0.87	0.0149	0.78	0.55-1.11	0.1717	0.44	0.25-0.78	0.0049
Age	1.00	0.98-1.02	0.8789	1.01	1.00-1.03	0.0711	1.00	0.98-1.02	0.9516
Sex: Female	0.66	0.37-1.18	0.1623	0.49	0.33-0.72	0.0003	0.61	0.35-1.09	0.0966
Ethnicity: Asian	1.17	0.46-2.94	0.7440	0.55	0.24-1.26	0.1571	0.23	0.03-1.64	0.1419
Ethnicity: Black	0.51	0.07-3.72	0.5098	0.94	0.35-2.56	0.9108	0.50	0.07-3.62	0.4935
Ethnicity: Mixed	N/A	N/A	N/A	N/A	N/A	N/A	N/A	N/A	N/A
Ethnicity: Other	N/A	N/A	N/A	1.29	0.32-5.23	0.7194	N/A	N/A	N/A
IMD	0.98	0.93-1.03	0.3770	1.01	0.97-1.04	0.7031	1.01	0.96-1.06	0.6934
North West	1.25	0.63-2.51	0.5198	1.29	0.84-1.99	0.2517	1.25	0.64-2.42	0.5133
Yorkshire & Humber	1.68	0.52-5.41	0.3814	1.27	0.56-2.89	0.5623	0.55	0.08-3.95	0.5492
East Midlands	1.97	0.48-8.11	0.3463	0.77	0.19-3.10	0.7104	2.70	0.84-8.65	0.0949
West Midlands	0.79	0.31-1.98	0.6125	1.13	0.67-1.90	0.6569	1.06	0.48-2.34	0.8869
East of England	1.09	0.46-2.55	0.8449	0.77	0.40-1.47	0.4334	1.25	0.57-2.77	0.5797
South West	1.18	0.56-2.51	0.6653	0.81	0.46-1.41	0.4595	1.47	0.74-2.93	0.2685
South Central	1.00	0.43-2.36	0.9911	0.98	0.57-1.68	0.9338	0.43	0.13-1.38	0.1564
London	0.39	0.12-1.25	0.1134	0.93	0.54-1.59	0.7863	0.52	0.19-1.45	0.2114
South East Coast	1.02	0.46-2.26	0.9653	0.98	0.58-1.66	0.9495	0.83	0.35-1.93	0.6570
BMI: Underweight	0.25	0.06-1.03	0.0552	N/A	N/A	N/A	0.61	0.24-1.53	0.2919
BMI: Overweight	0.75	0.39-1.43	0.3793	N/A	N/A	N/A	0.53	0.26-1.04	0.0660
BMI: Obese	1.74	0.97-3.12	0.0614	N/A	N/A	N/A	1.88	1.07-3.31	0.0285
BMI: Missing	1.54	0.38-6.35	0.5466	N/A	N/A	N/A	1.18	0.29-4.85	0.8162
Ex-Smoker	0.97	0.53-1.76	0.9086	1.39	0.97-1.99	0.0688	1.40	0.81-2.42	0.2292
Current Smoker	2.24	1.26-3.98	0.0059	1.47	0.94-2.32	0.0948	2.20	1.26-3.84	0.0057
Smoking: Missing	3.02	0.42-21.88	0.2746	1.27	0.18-9.13	0.8095	3.21	0.44-23.27	0.2483
Alcohol: 1-14	0.95	0.55-1.64	0.8405	1.01	0.71-1.44	0.9673	0.92	0.54-1.58	0.7723
Alcohol: 15-42	0.16	0.02-1.16	0.0694	0.65	0.32-1.33	0.2410	0.32	0.08-1.33	0.1178
Alcohol: >42	5.22	2.35-11.58	<0.0001	2.73	1.33-5.59	0.0060	2.85	1.13-7.15	0.0258
Alcohol: Missing	1.07	0.54-2.14	0.8470	0.96	0.59-1.57	0.8720	1.04	0.52-2.06	0.9202
Morbidities	1.18	1.01-1.38	0.0406	1.35	1.23-1.48	<0.0001	1.21	1.04-1.40	0.0117
Prescriptions	1.02	1.01-1.04	0.0066	1.02	1.01-1.03	<0.0001	1.02	1.00-1.04	0.0283
Hospitalisations	1.47	1.16-1.85	0.0012	1.49	1.31-1.69	<0.0001	1.44	1.22-1.70	<0.0001
Duration of diabetes (years)	1.02	0.98-1.07	0.2823	1.00	0.97-1.03	0.9531	1.02	0.98-1.07	0.3370
Complications	2.52	2.10-3.03	<0.0001	2.77	2.44-3.14	<0.0001	2.75	2.30-3.29	<0.0001
Glucose lowering therapies	1.42	1.11-1.82	0.0054	1.42	1.21-1.66	<0.0001	1.32	1.07-1.63	0.0109
Insulin prescription	3.37	1.85-6.16	0.0001	4.99	3.50-7.11	<0.0001	3.35	1.87-6.02	<0.0001

Study sizes across exposures after 1:1 propensity score matching are provided in Supplementary Table 5, as they are the same between univariate and multivariate analyses.

N/A indicates no observations for the covariate after propensity score matching.

Supplementary Table 3: Univariate hazard ratios (with corresponding 95% CIs and p-values) for minor or major amputation risk by each covariate across NDA and QOF exposure definitions after 1:1 propensity score matching.

	**Exposure Definition**								
	**Achieve All QOF Targets**			**Meet All NDA Processes**			**Achieve All QOF & NDA Targets**		
	**Hazard Ratio**	**95% CI**	**p**	**Hazard Ratio**	**95% CI**	**p**	**Hazard Ratio**	**95% CI**	**p**
Exposure	0.64	0.51-0.81	0.0002	0.56	0.46-0.67	<0.0001	0.49	0.35-0.68	<0.0001
Age	1.02	1.01-1.03	0.0032	1.01	1.01-1.02	0.0016	1.02	1.01-1.04	0.0022
Sex: Female	0.60	0.47-0.77	0.0001	0.52	0.42-0.64	<0.0001	0.61	0.44-0.87	0.0055
Ethnicity: Asian	0.38	0.18-0.81	0.0121	0.25	0.12-0.53	0.0003	0.24	0.06-0.98	0.0472
Ethnicity: Black	0.78	0.29-2.11	0.6302	1.07	0.55-2.07	0.8403	0.47	0.07-3.36	0.4524
Ethnicity: Mixed	0.98	0.24-3.95	0.9813	0.89	0.28-2.76	0.8351	N/A	N/A	N/A
Ethnicity: Other	0.56	0.14-2.25	0.4132	0.38	0.10-1.54	0.1753	0.57	0.08-4.08	0.5772
IMD	1.01	0.99-1.03	0.3759	1.01	1.00-1.03	0.1385	1.04	1.01-1.07	0.0152
North West	1.22	0.92-1.63	0.1656	1.25	0.99-1.58	0.0582	1.23	0.83-1.81	0.3037
Yorkshire & Humber	0.78	0.40-1.51	0.4526	0.65	0.36-1.19	0.1638	1.16	0.54-2.48	0.7021
East Midlands	1.12	0.53-2.38	0.7599	0.81	0.40-1.63	0.5501	1.19	0.44-3.21	0.7313
West Midlands	0.82	0.56-1.21	0.3242	0.71	0.51-1.00	0.0470	0.78	0.46-1.33	0.3660
East of England	0.85	0.57-1.28	0.4394	0.87	0.63-1.21	0.4060	0.97	0.58-1.63	0.9118
South West	1.34	0.99-1.83	0.0605	1.28	0.99-1.65	0.0548	0.99	0.63-1.57	0.9779
South Central	0.92	0.64-1.33	0.6720	1.11	0.84-1.46	0.4777	1.25	0.80-1.97	0.3269
London	1.12	0.81-1.55	0.5021	1.09	0.83-1.43	0.5230	1.00	0.63-1.60	0.9960
South East Coast	0.67	0.44-1.01	0.0551	0.80	0.59-1.09	0.1623	0.63	0.35-1.14	0.1309
BMI: Underweight	1.25	0.93-1.67	0.1430	1.12	0.86-1.47	0.3892	1.10	0.72-1.68	0.6560
BMI: Overweight	0.97	0.76-1.24	0.8181	0.93	0.76-1.14	0.4821	0.93	0.67-1.30	0.6801
BMI: Obese	0.84	0.67-1.07	0.1571	0.99	0.82-1.19	0.8938	0.92	0.67-1.26	0.6028
BMI: Missing	1.96	0.73-5.25	0.1827	1.40	0.58-3.37	0.4576	2.93	0.93-9.17	0.0656
Ex-Smoker	1.36	1.08-1.72	0.0091	1.27	1.05-1.54	0.0126	1.38	1.01-1.90	0.0445
Current Smoker	1.45	1.07-1.97	0.0169	1.45	1.14-1.85	0.0028	1.49	0.98-2.25	0.0612
Smoking: Missing	3.08	0.77-12.40	0.1127	N/A	N/A	N/A	N/A	N/A	N/A
Alcohol: 1-14	0.79	0.62-0.99	0.0434	0.86	0.71-1.04	0.1124	0.83	0.60-1.14	0.2502
Alcohol: 15-42	1.01	0.69-1.49	0.9406	1.08	0.80-1.47	0.6040	0.86	0.50-1.49	0.5870
Alcohol: >42	1.49	0.74-3.00	0.2684	1.56	0.90-2.71	0.1148	1.11	0.36-3.49	0.8524
Alcohol: Missing	1.33	0.96-1.84	0.0825	0.89	0.65-1.23	0.4783	1.08	0.64-1.81	0.7827
Morbidities	1.33	1.26-1.42	<0.0001	1.30	1.24-1.37	<0.0001	1.30	1.20-1.42	<0.0001
Prescriptions	1.03	1.02-1.03	<0.0001	1.02	1.02-1.03	<0.0001	1.02	1.01-1.03	0.0062
Hospitalisations	1.67	1.51-1.83	<0.0001	1.60	1.46-1.75	<0.0001	1.52	1.19-1.95	0.0009
Duration of diabetes (years)	1.02	1.00-1.04	0.0258	1.03	1.02-1.05	<0.0001	1.02	1.00-1.05	0.0754
Complications	2.52	2.33-2.74	<0.0001	2.63	2.46-2.80	<0.0001	2.41	2.15-2.69	<0.0001
Glucose lowering therapies	1.33	1.19-1.49	<0.0001	1.32	1.21-1.44	<0.0001	1.31	1.12-1.53	0.0010
Insulin prescription	4.60	3.56-5.94	<0.0001	4.18	3.44-5.07	<0.0001	4.63	3.23-6.65	<0.0001

Study sizes across exposures after 1:1 propensity score matching are provided in Supplementary Table 6, as they are the same between univariate and multivariate analyses.

N/A indicates no observations for the covariate after propensity score matching.

Supplementary Table 4: Multivariate hazard ratios (with corresponding 95% CIs and p-values) for minor or major amputation risk by each covariate across QOF exposure definitions after 1:1 propensity score matching, including the adjusted study size (n) and C-statistic (also with corresponding 95% CI).

	**Exposure Definition**								
	**Achieve HbA1c QOF Target**			**Achieve Blood Pressure QOF Target**			**Achieve Cholesterol QOF Target**		
	**Hazard Ratio**	**95% CI**	**p**	**Hazard Ratio**	**95% CI**	**p**	**Hazard Ratio**	**95% CI**	**p**
Exposure	0.61	0.49-0.74	<0.0001	0.88	0.73-1.06	0.1891	0.67	0.53-0.86	0.0017
Age	0.99	0.98-1.00	0.0428	0.98	0.97-0.99	0.0003	0.99	0.97-1.00	0.0317
Sex: Female	0.91	0.71-1.16	0.4334	0.99	0.80-1.24	0.9632	0.95	0.71-1.26	0.7175
Ethnicity: Asian	0.24	0.12-0.47	<0.0001	0.31	0.17-0.56	0.0001	0.44	0.22-0.87	0.0187
Ethnicity: Black	0.92	0.47-1.79	0.7957	0.92	0.50-1.68	0.7743	0.69	0.28-1.68	0.4118
Ethnicity: Mixed	0.85	0.27-2.67	0.7860	0.76	0.24-2.37	0.6325	0.45	0.06-3.23	0.4279
Ethnicity: Other	0.44	0.11-1.77	0.2474	0.34	0.09-1.38	0.1325	0.29	0.04-2.11	0.2236
IMD	1.00	0.98-1.02	0.7579	1.01	0.99-1.02	0.4542	1.02	1.00-1.04	0.0765
North West	1.22	0.63-2.36	0.5614	1.32	0.69-2.54	0.4052	1.45	0.62-3.39	0.3917
Yorkshire & Humber	1.13	0.51-2.50	0.7665	0.70	0.30-1.66	0.4201	0.98	0.33-2.95	0.9757
East Midlands	0.83	0.32-2.18	0.7048	1.15	0.48-2.78	0.7482	1.33	0.43-4.16	0.6197
West Midlands	0.78	0.38-1.58	0.4907	0.89	0.44-1.78	0.7368	0.95	0.38-2.34	0.9037
East of England	0.99	0.49-1.99	0.9701	1.11	0.56-2.22	0.7641	1.63	0.67-3.95	0.2817
South West	1.22	0.62-2.39	0.5697	1.41	0.72-2.73	0.3142	1.58	0.67-3.74	0.2977
South Central	1.17	0.59-2.33	0.6453	1.30	0.66-2.57	0.4477	1.47	0.61-3.56	0.3929
London	1.13	0.58-2.21	0.7267	1.24	0.64-2.41	0.5298	1.28	0.54-3.07	0.5729
South East Coast	0.94	0.47-1.87	0.8509	0.98	0.49-1.96	0.9637	1.25	0.52-3.05	0.6163
BMI: Underweight	0.89	0.28-2.85	0.8437	0.63	0.25-1.56	0.3166	1.12	0.27-4.66	0.8735
BMI: Overweight	0.65	0.21-2.05	0.4615	0.47	0.19-1.15	0.0981	0.90	0.22-3.67	0.8811
BMI: Obese	0.64	0.21-2.02	0.4507	0.45	0.18-1.10	0.0803	0.88	0.21-3.56	0.8521
BMI: Missing	0.80	0.20-3.22	0.7546	0.52	0.16-1.70	0.2774	1.10	0.20-6.05	0.9134
Ex-Smoker	1.03	0.82-1.30	0.7865	0.95	0.76-1.18	0.6234	1.14	0.85-1.52	0.3885
Current Smoker	1.30	0.98-1.74	0.0712	1.36	1.04-1.77	0.0235	1.48	1.06-2.07	0.0226
Smoking: Missing	2.74	0.67-11.10	0.1589	2.00	0.49-8.06	0.3322	2.08	0.29-15.01	0.4670
Alcohol: 1-14	0.83	0.63-1.09	0.1761	0.84	0.65-1.08	0.1683	0.92	0.65-1.30	0.6467
Alcohol: 15-42	0.93	0.62-1.40	0.7364	0.82	0.56-1.21	0.3168	0.87	0.52-1.45	0.6039
Alcohol: >42	1.91	1.14-3.18	0.0135	1.36	0.82-2.26	0.2314	2.33	1.31-4.13	0.0039
Alcohol: Missing	1.31	0.92-1.86	0.1302	1.21	0.87-1.69	0.2537	1.29	0.83-2.00	0.2525
Morbidities	0.88	0.82-0.94	0.0001	0.87	0.82-0.93	<0.0001	0.86	0.79-0.93	0.0002
Prescriptions	0.99	0.98-1.00	0.1677	1.00	0.99-1.01	0.7749	0.99	0.98-1.01	0.2138
Hospitalisations	1.25	1.13-1.38	<0.0001	1.22	1.11-1.35	0.0001	1.12	0.96-1.30	0.1412
Duration of diabetes (years)	1.01	0.99-1.02	0.4805	1.01	0.99-1.03	0.2039	1.01	0.99-1.03	0.1861
Complications	2.70	2.49-2.94	<0.0001	2.80	2.59-3.03	<0.0001	2.89	2.62-3.20	<0.0001
Glucose lowering therapies	1.02	0.90-1.15	0.7839	1.04	0.93-1.16	0.5031	1.09	0.95-1.26	0.2297
Insulin prescription	1.78	1.43-2.21	<0.0001	1.80	1.45-2.23	<0.0001	1.47	1.10-1.96	0.0093
	**Value**	**95% CI (lower)**	**95% CI (upper)**	**Value**	**95% CI (lower)**	**95% CI (upper)**	**Value**	**95% CI (lower)**	**95% CI (upper)**
n after matching	53,310			69,974			41,254		
C-statistic	0.8691	0.8689	0.8693	0.8620	0.8618	0.8621	0.8729	0.8726	0.8732

Reference groups for categorical covariates include: white (ethnicity); North East (region); normal weight (BMI); non-smoker (smoking status); 0 units (alcohol consumption); and no insulin prescription (insulin use).

Supplementary Table 5: Multivariate hazard ratios (with corresponding 95% CIs and p-values) for minor or major amputation risk by each covariate across NDA exposure definitions after 1:1 propensity score matching, including the adjusted study size (n) and C-statistic (also with corresponding 95% CI).

	**Exposure Definition**								
	**Meet 4-6 NDA Processes (vs. Meet 0-3 NDA Processes)**			**Meet 7-9 NDA Processes (vs. Meet 0-3 NDA Processes)**			**Meet 7-9 NDA Processes (vs. Meet 4-6 NDA Processes)**		
	**Hazard Ratio**	**95% CI**	**p**	**Hazard Ratio**	**95% CI**	**p**	**Hazard Ratio**	**95% CI**	**p**
Exposure	0.45	0.24-0.83	0.0106	0.67	0.47-0.97	0.0319	0.38	0.20-0.70	0.0022
Age	1.00	0.97-1.03	0.9866	0.99	0.97-1.01	0.2824	0.99	0.97-1.02	0.6379
Sex: Female	1.13	0.58-2.19	0.7266	0.79	0.51-1.23	0.3018	1.16	0.60-2.23	0.6639
Ethnicity: Asian	2.06	0.74-5.75	0.1670	0.60	0.25-1.42	0.2429	0.41	0.05-3.12	0.3880
Ethnicity: Black	0.86	0.11-6.55	0.8832	1.39	0.51-3.84	0.5200	0.78	0.10-5.91	0.8105
Ethnicity: Mixed	N/A	N/A	N/A	N/A	N/A	N/A	N/A	N/A	N/A
Ethnicity: Other	N/A	N/A	N/A	1.77	0.42-7.41	0.4315	N/A	N/A	N/A
IMD	0.97	0.92-1.03	0.2876	1.01	0.98-1.04	0.5767	1.00	0.94-1.05	0.8831
North West	1.15	0.14-9.39	0.8947	1.23	0.37-4.09	0.7362	0.75	0.16-3.52	0.7147
Yorkshire & Humber	1.47	0.14-5.05	0.7453	1.26	0.31-5.06	0.7492	0.38	0.03-4.44	0.4441
East Midlands	0.96	0.08-11.37	0.9760	0.57	0.09-3.48	0.5434	1.60	0.25-10.23	0.6205
West Midlands	0.70	0.08-6.33	0.7494	1.15	0.33-3.99	0.8269	0.84	0.17-4.26	0.8338
East of England	1.09	0.12-9.51	0.9387	0.75	0.20-2.76	0.6600	0.89	0.17-4.59	0.8919
South West	1.00	0.12-8.33	0.9964	0.80	0.23-2.84	0.7357	0.95	0.20-4.50	0.9517
South Central	0.62	0.07-5.46	0.6644	0.97	0.27-3.43	0.9616	0.24	0.04-1.55	0.1334
London	0.33	0.03-3.48	0.3530	0.98	0.28-3.43	0.9748	0.33	0.06-1.90	0.2129
South East Coast	0.76	0.09-6.69	0.8078	0.92	0.26-3.23	0.8988	0.47	0.09-2.55	0.3793
BMI: Underweight	0.31	0.03-3.79	0.3580	N/A	N/A	N/A	0.48	0.05-4.61	0.5252
BMI: Overweight	0.98	0.11-8.35	0.9816	N/A	N/A	N/A	0.50	0.06-4.32	0.5320
BMI: Obese	1.99	0.24-16.41	0.5227	N/A	N/A	N/A	1.35	0.17-10.91	0.7757
BMI: Missing	1.64	0.13-20.33	0.6999	N/A	N/A	N/A	1.09	0.09-13.73	0.9467
Ex-Smoker	1.04	0.49-2.19	0.9175	1.11	0.72-1.69	0.6445	1.70	0.81-3.57	0.1619
Current Smoker	2.64	1.27-5.49	0.0092	1.41	0.83-2.39	0.2011	3.36	1.56-7.26	0.0020
Smoking: Missing	6.99	0.87-56.14	0.0674	2.65	0.36-19.55	0.3404	6.82	0.83-55.79	0.0733
Alcohol: 1-14	1.19	0.50-2.80	0.6947	1.11	0.66-1.88	0.6834	0.89	0.40-2.00	0.7854
Alcohol: 15-42	0.20	0.02-1.71	0.1421	0.85	0.36-1.99	0.7092	0.27	0.06-1.34	0.1092
Alcohol: >42	6.45	1.95-21.39	0.0023	2.28	0.96-5.46	0.0633	2.56	0.76-8.69	0.1310
Alcohol: Missing	1.69	0.62-4.60	0.3033	1.38	0.72-2.62	0.3283	1.41	0.55-3.62	0.4801
Morbidities	0.77	0.62-0.95	0.0133	0.91	0.81-1.02	0.1150	0.81	0.66-0.98	0.0313
Prescriptions	0.99	0.96-1.02	0.3627	1.00	0.98-1.02	0.9766	0.98	0.95-1.01	0.1065
Hospitalisations	1.23	0.87-1.73	0.2347	1.15	0.97-1.37	0.1100	1.31	1.07-1.60	0.0087
Duration of diabetes (years)	1.02	0.97-1.07	0.4354	0.98	0.94-1.01	0.1783	1.01	0.96-1.06	0.7652
Complications	3.00	2.36-3.81	<0.0001	2.82	2.41-3.29	<0.0001	3.20	2.56-4.01	<0.0001
Glucose lowering therapies	1.16	0.83-1.63	0.3878	1.11	0.90-1.36	0.3257	1.24	0.91-1.70	0.1746
Insulin prescription	1.70	0.83-3.49	0.1459	2.20	1.47-3.30	0.0001	1.67	0.85-3.28	0.1402
	**Value**	**95% CI (lower)**	**95% CI (upper)**	**Value**	**95% CI (lower)**	**95% CI (upper)**	**Value**	**95% CI (lower)**	**95% CI (upper)**
n after matching	6,820			19,074			6,892		
C-statistic	0.8996	0.8983	0.9008	0.8859	0.8854	0.8864	0.9260	0.9255	0.9264

Reference groups for categorical covariates include: white (ethnicity); North East (region); normal weight (BMI); non-smoker (smoking status); 0 units (alcohol consumption); and no insulin prescription (insulin use).
N/A indicates no observations for the covariate after propensity score matching.

Supplementary Table 6: Multivariate hazard ratios (with corresponding 95% CIs and p-values) for minor or major amputation risk by each covariate across QOF and NDA exposure definitions after 1:1 propensity score matching, including the adjusted study size (n) and C-statistic (also with corresponding 95% CI).

	**Exposure Definition**								
	**Achieve All QOF Targets**			**Meet All NDA Processes**			**Achieve All QOF & NDA Targets**		
	**Hazard Ratio**	**95% CI**	**p**	**Hazard Ratio**	**95% CI**	**p**	**Hazard Ratio**	**95% CI**	**p**
Exposure	0.64	0.50-0.81	0.0002	0.58	0.48-0.71	<0.0001	0.49	0.35-0.68	<0.0001
Age	0.99	0.97-1.00	0.0434	0.99	0.98-1.00	0.0475	1.00	0.98-1.02	0.9591
Sex: Female	1.12	0.85-1.47	0.4361	0.95	0.75-1.19	0.6292	1.01	0.69-1.47	0.9552
Ethnicity: Asian	0.31	0.14-0.67	0.0031	0.22	0.10-0.47	0.0001	0.21	0.05-0.87	0.0316
Ethnicity: Black	0.86	0.32-2.33	0.7687	1.13	0.58-2.19	0.7257	0.51	0.07-3.65	0.5009
Ethnicity: Mixed	0.85	0.21-3.46	0.8244	0.85	0.27-2.67	0.7875	N/A	N/A	N/A
Ethnicity: Other	0.60	0.15-2.45	0.4790	0.39	0.10-1.59	0.1899	0.65	0.09-4.74	0.6745
IMD	1.01	0.98-1.03	0.6459	1.01	0.99-1.03	0.3340	1.04	1.01-1.07	0.0133
North West	1.06	0.51-2.22	0.8802	1.47	0.74-2.92	0.2717	1.28	0.45-3.63	0.6445
Yorkshire & Humber	0.63	0.24-1.64	0.3453	0.78	0.32-1.89	0.5814	1.10	0.32-3.78	0.8817
East Midlands	1.00	0.36-2.77	0.9977	0.91	0.35-2.36	0.8439	1.42	0.35-5.74	0.6200
West Midlands	0.71	0.33-1.57	0.4023	0.91	0.44-1.89	0.7989	0.90	0.30-2.75	0.8599
East of England	0.76	0.34-1.68	0.4946	1.10	0.53-2.27	0.8073	1.17	0.39-3.56	0.7803
South West	1.08	0.51-2.28	0.8496	1.50	0.75-3.00	0.2567	1.12	0.38-3.31	0.8326
South Central	0.82	0.38-1.81	0.6286	1.36	0.67-2.76	0.4014	1.56	0.53-4.60	0.4221
London	0.97	0.45-2.08	0.9409	1.29	0.64-2.59	0.4837	1.03	0.35-3.05	0.9534
South East Coast	0.62	0.28-1.39	0.2440	1.01	0.49-2.09	0.9708	0.86	0.27-2.71	0.7931
BMI: Underweight	0.49	0.19-1.22	0.1243	0.69	0.25-1.90	0.4696	0.44	0.13-1.46	0.1790
BMI: Overweight	0.36	0.14-0.88	0.0253	0.53	0.20-1.44	0.2135	0.39	0.12-1.26	0.1167
BMI: Obese	0.30	0.12-0.75	0.0098	0.51	0.19-1.37	0.1819	0.37	0.11-1.20	0.0990
BMI: Missing	0.62	0.17-2.33	0.4815	0.65	0.17-2.42	0.5155	1.15	0.23-5.81	0.8667
Ex-Smoker	1.14	0.87-1.50	0.3330	1.01	0.81-1.26	0.9303	1.16	0.80-1.68	0.4468
Current Smoker	1.51	1.06-2.15	0.0238	1.32	1.00-1.75	0.0533	1.65	1.01-2.69	0.0452
Smoking: Missing	3.82	0.94-15.58	0.0613	N/A	N/A	N/A	N/A	N/A	N/A
Alcohol: 1-14	0.84	0.61-1.16	0.2819	0.84	0.65-1.09	0.1887	0.78	0.51-1.18	0.2368
Alcohol: 15-42	0.94	0.58-1.51	0.7948	0.95	0.65-1.38	0.7939	0.71	0.37-1.37	0.3092
Alcohol: >42	1.17	0.53-2.55	0.6988	1.09	0.60-2.00	0.7695	0.82	0.24-2.73	0.7416
Alcohol: Missing	1.55	1.03-2.34	0.0372	1.04	0.71-1.51	0.8454	1.24	0.67-2.28	0.4931
Morbidities	0.90	0.83-0.97	0.0056	0.87	0.82-0.93	<0.0001	0.91	0.81-1.02	0.0904
Prescriptions	1.00	0.99-1.01	0.8868	1.00	0.98-1.01	0.4498	0.99	0.96-1.01	0.3491
Hospitalisations	1.31	1.16-1.48	<0.0001	1.23	1.09-1.38	0.0005	1.13	0.85-1.50	0.3989
Duration of diabetes (years)	1.00	0.98-1.02	0.8483	1.01	1.00-1.03	0.1854	1.01	0.98-1.03	0.6786
Complications	2.61	2.36-2.88	<0.0001	2.70	2.49-2.92	<0.0001	2.42	2.10-2.78	<0.0001
Glucose lowering therapies	1.06	0.92-1.22	0.4233	1.08	0.97-1.21	0.1747	1.12	0.91-1.37	0.3042
Insulin prescription	2.25	1.69-2.99	<0.0001	1.94	1.55-2.41	<0.0001	2.41	1.61-3.62	<0.0001
	**Value**	**95% CI (lower)**	**95% CI (upper)**	**Value**	**95% CI (lower)**	**95% CI (upper)**	**Value**	**95% CI (lower)**	**95% CI (upper)**
n after matching	55,306			70,924			28,128		
C-statistic	0.8409	0.8405	0.8413	0.8589	0.8586	0.8591	0.8442	0.8435	0.8448

Reference groups for categorical covariates include: white (ethnicity); North East (region); normal weight (BMI); non-smoker (smoking status); 0 units (alcohol consumption); and no insulin prescription (insulin use).

N/A indicates no observations for the covariate after propensity score matching.

Supplementary Table 7: Univariate hazard ratios (with corresponding 95% CIs and p-values) for minor or major amputation risk by each covariate across QOF exposure definitions, *among those who meet all other QOF targets*, after 1:1 propensity score matching.

	**Exposure Definition**								
	**Achieve HbA1c QOF Target**			**Achieve Blood Pressure QOF Target**			**Achieve Cholesterol QOF Target**		
	**Hazard Ratio**	**95% CI**	**p**	**Hazard Ratio**	**95% CI**	**p**	**Hazard Ratio**	**95% CI**	**p**
Exposure	0.78	0.57-1.06	0.1090	0.99	0.71-1.38	0.9336	1.09	0.63-1.89	0.7505
Age	1.02	1.00-1.03	0.0078	1.03	1.01-1.04	0.0010	1.03	1.01-1.06	0.0183
Sex: Female	0.46	0.32-0.65	<0.0001	0.63	0.44-0.91	0.0130	0.57	0.32-0.99	0.0457
Ethnicity: Asian	0.26	0.09-0.69	0.0070	0.34	0.11-1.06	0.0638	N/A	N/A	N/A
Ethnicity: Black	0.86	0.27-2.68	0.7894	1.34	0.50-3.63	0.5604	0.92	0.13-6.68	0.9366
Ethnicity: Mixed	0.76	0.11-5.44	0.7860	0.96	0.13-6.88	0.9698	N/A	N/A	N/A
Ethnicity: Other	0.46	0.06-3.25	0.4333	N/A	N/A	N/A	N/A	N/A	N/A
IMD	0.99	0.97-1.02	0.6017	1.00	0.97-1.04	0.7579	1.04	0.99-1.10	0.0840
North West	1.12	0.76-1.65	0.5791	1.09	0.71-1.67	0.6903	1.96	1.07-3.58	0.0283
Yorkshire & Humber	1.44	0.73-2.81	0.2910	0.72	0.27-1.95	0.5205	0.50	0.07-3.65	0.4976
East Midlands	0.76	0.24-2.39	0.6425	0.97	0.31-3.04	0.9552	N/A	N/A	N/A
West Midlands	0.71	0.41-1.23	0.2267	0.53	0.27-1.04	0.0661	0.80	0.32-2.02	0.6416
East of England	0.79	0.46-1.37	0.4071	0.68	0.36-1.30	0.2411	0.99	0.39-2.49	0.9823
South West	1.37	0.91-2.04	0.1283	1.62	1.07-2.45	0.0229	1.20	0.56-2.54	0.6415
South Central	0.99	0.62-1.58	0.9665	0.65	0.35-1.20	0.1685	0.60	0.22-1.68	0.3337
London	0.98	0.62-1.55	0.9275	1.34	0.85-2.09	0.2024	1.20	0.56-2.55	0.6361
South East Coast	0.93	0.58-1.50	0.7611	1.32	0.83-2.08	0.2371	0.44	0.14-1.40	0.1638
BMI: Underweight	1.20	0.79-1.83	0.3838	1.79	1.21-2.66	0.0039	1.76	0.95-3.26	0.0711
BMI: Overweight	1.05	0.76-1.45	0.7731	1.06	0.75-1.50	0.7545	1.04	0.58-1.84	0.9008
BMI: Obese	0.79	0.58-1.07	0.1319	0.69	0.49-0.97	0.0349	0.53	0.29-0.96	0.0351
BMI: Missing	2.45	0.91-6.61	0.0768	N/A	N/A	N/A	5.44	1.32-22.36	0.0189
Ex-Smoker	1.23	0.90-1.68	0.1894	1.31	0.94-1.84	0.1129	1.13	0.64-1.98	0.6759
Current Smoker	1.47	0.99-2.18	0.0586	1.42	0.91-2.22	0.1256	1.37	0.66-2.80	0.3966
Smoking: Missing	N/A	N/A	N/A	N/A	N/A	N/A	N/A	N/A	N/A
Alcohol: 1-14	0.81	0.60-1.10	0.1797	0.70	0.50-0.97	0.0342	0.74	0.43-1.28	0.2829
Alcohol: 15-42	1.03	0.60-1.79	0.9102	0.93	0.53-1.61	0.7901	1.24	0.53-2.91	0.6190
Alcohol: >42	0.78	0.19-3.15	0.7287	2.20	0.97-4.98	0.0592	1.91	0.46-7.85	0.3705
Alcohol: Missing	1.00	0.64-1.55	0.9999	1.30	0.81-2.08	0.2853	0.77	0.31-1.95	0.5853
Morbidities	1.28	1.18-1.38	<0.0001	1.38	1.26-1.50	<0.0001	1.49	1.29-1.71	<0.0001
Prescriptions	1.02	1.00-1.03	0.0088	1.02	1.01-1.03	0.0010	1.04	1.02-1.05	<0.0001
Hospitalisations	1.62	1.45-1.82	<0.0001	1.79	1.61-2.00	<0.0001	1.50	1.12-2.01	0.0070
Duration of diabetes (years)	1.03	1.01-1.05	0.0133	1.01	0.99-1.04	0.3131	1.04	0.99-1.08	0.0930
Complications	2.55	2.29-2.84	<0.0001	2.49	2.21-2.79	<0.0001	2.95	2.44-3.58	<0.0001
Glucose lowering therapies	1.05	0.89-1.23	0.5675	1.18	0.99-1.41	0.0617	1.51	1.17-1.95	0.0018
Insulin prescription	3.12	2.29-4.24	<0.0001	4.75	3.28-6.88	<0.0001	7.71	4.39-13.53	<0.0001

Study sizes across exposures after 1:1 propensity score matching are provided in Supplementary Table 8, as they are the same between univariate and multivariate analyses.

N/A indicates no observations for the covariate after propensity score matching.

Supplementary Table 8: Multivariate hazard ratios (with corresponding 95% CIs and p-values) for minor or major amputation risk by each covariate across QOF exposure definitions, *among those who meet all other QOF targets*, after 1:1 propensity score matching, including the adjusted study size (n) and C-statistic (also with corresponding 95% CI).

	**Exposure Definition**								
	**Achieve HbA1c QOF Target**			**Achieve Blood Pressure QOF Target**			**Achieve Cholesterol QOF Target**		
	**Hazard Ratio**	**95% CI**	**p**	**Hazard Ratio**	**95% CI**	**p**	**Hazard Ratio**	**95% CI**	**p**
Exposure	0.83	0.61-1.13	0.2399	0.92	0.65-1.29	0.6240	1.04	0.59-1.84	0.8816
Age	0.99	0.97-1.00	0.1352	0.99	0.97-1.01	0.4343	0.99	0.96-1.02	0.6499
Sex: Female	0.82	0.55-1.21	0.3140	0.99	0.66-1.47	0.9453	1.02	0.53-1.94	0.9572
Ethnicity: Asian	0.19	0.07-0.54	0.0016	0.28	0.09-0.93	0.0369	N/A	N/A	N/A
Ethnicity: Black	0.77	0.24-2.49	0.6684	1.56	0.57-4.30	0.3855	0.88	0.12-6.60	0.9044
Ethnicity: Mixed	0.84	0.12-6.09	0.8657	0.68	0.09-4.97	0.7041	N/A	N/A	N/A
Ethnicity: Other	0.49	0.07-3.53	0.4755	N/A	N/A	N/A	N/A	N/A	N/A
IMD	0.99	0.96-1.02	0.6050	1.00	0.97-1.03	0.9482	1.02	0.97-1.08	0.3883
North West	1.47	0.45-4.83	0.5253	3.44	0.47-25.44	0.2254	0.90	0.20-4.09	0.8919
Yorkshire & Humber	1.78	0.48-6.64	0.3879	2.36	0.26-21.19	0.4440	0.36	0.03-4.07	0.4071
East Midlands	0.92	0.18-4.61	0.9194	2.83	0.28-28.22	0.3763	N/A	N/A	N/A
West Midlands	0.83	0.24-2.89	0.7637	1.86	0.24-14.77	0.5558	0.58	0.11-3.14	0.5291
East of England	1.03	0.29-3.61	0.9645	2.41	0.31-19.00	0.4035	0.86	0.16-4.69	0.8608
South West	1.59	0.48-5.26	0.4451	4.80	0.65-35.50	0.1240	0.79	0.16-3.90	0.7711
South Central	1.16	0.34-3.95	0.8148	2.37	0.30-18.55	0.4113	0.47	0.08-2.72	0.3962
London	1.26	0.37-4.25	0.7088	4.07	0.55-30.26	0.1700	0.83	0.17-4.04	0.8165
South East Coast	1.19	0.35-4.08	0.7801	4.08	0.54-30.53	0.1712	0.30	0.05-1.92	0.2053
BMI: Underweight	0.38	0.11-1.27	0.1171	1.42	0.19-10.48	0.7314	0.65	0.08-5.13	0.6788
BMI: Overweight	0.28	0.09-0.92	0.0353	0.90	0.12-6.60	0.9176	0.32	0.04-2.53	0.2819
BMI: Obese	0.23	0.07-0.73	0.0130	0.67	0.09-4.91	0.6906	0.17	0.02-1.41	0.1012
BMI: Missing	0.86	0.19-3.92	0.8504	N/A	N/A	N/A	1.25	0.11-14.79	0.8580
Ex-Smoker	0.92	0.64-1.32	0.6494	1.08	0.73-1.59	0.7081	0.67	0.35-1.29	0.2292
Current Smoker	1.25	0.79-1.97	0.3368	1.43	0.85-2.38	0.1763	1.09	0.48-2.46	0.8434
Smoking: Missing	N/A	N/A	N/A	N/A	N/A	N/A	N/A	N/A	N/A
Alcohol: 1-14	0.65	0.44-0.97	0.0335	0.69	0.44-1.09	0.1108	0.63	0.30-1.31	0.2128
Alcohol: 15-42	0.74	0.39-1.39	0.3514	0.73	0.37-1.43	0.3612	0.78	0.26-2.37	0.6626
Alcohol: >42	0.42	0.10-1.79	0.2415	1.73	0.70-4.31	0.2385	1.29	0.26-6.29	0.7555
Alcohol: Missing	1.17	0.69-1.98	0.5636	1.20	0.67-2.15	0.5437	0.87	0.30-2.56	0.8021
Morbidities	0.82	0.74-0.92	0.0004	0.89	0.79-1.00	0.0589	0.96	0.80-1.14	0.6164
Prescriptions	1.00	0.98-1.02	0.8167	1.00	0.98-1.02	0.8877	1.01	0.98-1.04	0.6859
Hospitalisations	1.36	1.17-1.57	0.0001	1.44	1.25-1.66	<0.0001	1.17	0.79-1.73	0.4344
Duration of diabetes (years)	1.01	0.98-1.03	0.4605	0.99	0.96-1.02	0.7071	1.01	0.96-1.06	0.7307
Complications	2.76	2.41-3.15	<0.0001	2.44	2.11-2.83	<0.0001	2.75	2.15-3.52	<0.0001
Glucose lowering therapies	1.01	0.83-1.23	0.9030	0.95	0.76-1.18	0.6490	1.08	0.75-1.54	0.6833
Insulin prescription	1.84	1.32-2.56	0.0003	2.60	1.69-4.00	<0.0001	3.50	1.77-6.93	0.0003
	**Value**	**95% CI (lower)**	**95% CI (upper)**	**Value**	**95% CI (lower)**	**95% CI (upper)**	**Value**	**95% CI (lower)**	**95% CI (upper)**
n after matching	20,620			32,430			11,802		
C-statistic	0.8565	0.8560	0.8571	0.8484	0.8477	0.8491	0.9118	0.9110	0.9126

Reference groups for categorical covariates include: white (ethnicity); North East (region); normal weight (BMI); non-smoker (smoking status); 0 units (alcohol consumption); and no insulin prescription (insulin use).

N/A indicates no observations for the covariate after propensity score matching.

Supplementary Table 9: Univariate hazard ratios (with corresponding 95% CIs and p-values) for minor or major amputation risk by each covariate across QOF exposure definitions, *among those who do not meet either of the other two QOF targets*, after 1:1 propensity score matching.

	Exposure Definition										
	Achieve HbA1c QOF Target				Achieve Blood Pressure QOF Target				Achieve Cholesterol QOF Target		
	Hazard Ratio	95% CI	p		Hazard Ratio	95% CI	p		Hazard Ratio	95% CI	p
Exposure	0.36	0.20-0.67	0.0012		0.76	0.51-1.13	0.1740		0.62	0.42-0.91	0.0144
Age	1.02	0.99-1.04	0.1331		1.01	1.00-1.03	0.0941		1.00	0.99-1.02	0.7114
Sex: Female	0.59	0.33-1.03	0.0624		0.53	0.36-0.80	0.0025		0.69	0.46-1.02	0.0627
Ethnicity: Asian	N/A	N/A	N/A		0.85	0.37-1.94	0.6959		0.34	0.11-1.07	0.0643
Ethnicity: Black	N/A	N/A	N/A		0.28	0.04-2.03	0.2087		0.99	0.36-2.69	0.9853
Ethnicity: Mixed	N/A	N/A	N/A		N/A	N/A	N/A		1.03	0.14-7.39	0.9755
Ethnicity: Other	1.31	0.18-9.51	0.7873		0.62	0.09-4.45	0.6359		0.65	0.09-4.64	0.6653
IMD	1.06	1.01-1.11	0.0253		1.01	0.98-1.05	0.4787		1.03	1.00-1.06	0.0934
North West	1.62	0.86-3.04	0.1325		1.19	0.73-1.93	0.4946		1.09	0.67-1.79	0.7252
Yorkshire & Humber	1.04	0.25-4.28	0.9570		0.89	0.28-2.81	0.8446		0.93	0.34-2.52	0.8847
East Midlands	0.95	0.13-6.91	0.9629		1.30	0.41-4.12	0.6499		1.38	0.44-4.36	0.5792
West Midlands	0.15	0.02-1.10	0.0622		0.85	0.46-1.60	0.6207		0.56	0.27-1.15	0.1149
East of England	1.16	0.50-2.73	0.7269		0.87	0.44-1.72	0.6817		1.13	0.62-2.05	0.6956
South West	0.82	0.35-1.92	0.6487		1.19	0.70-2.03	0.5276		1.26	0.76-2.09	0.3691
South Central	1.75	0.88-3.50	0.1113		1.08	0.60-1.94	0.7900		0.92	0.51-1.63	0.7648
London	0.88	0.37-2.05	0.7608		0.93	0.52-1.66	0.8019		1.15	0.68-1.96	0.5967
South East Coast	0.93	0.40-2.17	0.8596		0.70	0.36-1.35	0.2856		0.93	0.52-1.66	0.8057
BMI: Underweight	0.84	0.33-2.11	0.7105		1.16	0.67-2.01	0.5892		1.20	0.66-2.19	0.5450
BMI: Overweight	0.95	0.52-1.74	0.8693		0.89	0.58-1.36	0.5946		0.73	0.46-1.15	0.1719
BMI: Obese	1.03	0.59-1.79	0.9219		1.03	0.70-1.52	0.8721		1.15	0.78-1.71	0.4741
BMI: Missing	3.13	0.76-12.86	0.1142		0.84	0.12-6.00	0.8590		1.39	0.34-5.62	0.6462
Ex-Smoker	1.23	0.70-2.16	0.4805		1.51	1.02-2.24	0.0415		0.87	0.58-1.32	0.5217
Current Smoker	1.59	0.83-3.04	0.1605		1.12	0.70-1.80	0.6288		2.34	1.56-3.50	<0.0001
Smoking: Missing	N/A	N/A	N/A		N/A	N/A	N/A		N/A	N/A	N/A
Alcohol: 1-14	1.21	0.69-2.12	0.5109		0.95	0.64-1.41	0.8048		0.87	0.59-1.26	0.4548
Alcohol: 15-42	0.32	0.08-1.33	0.1179		1.33	0.71-2.48	0.3729		1.17	0.64-2.13	0.6085
Alcohol: >42	2.46	0.89-6.82	0.0843		2.27	0.84-6.17	0.1081		2.47	1.08-5.62	0.0315
Alcohol: Missing	1.39	0.68-2.85	0.3719		1.20	0.72-2.00	0.4845		1.14	0.69-1.87	0.6124
Morbidities	1.26	1.08-1.48	0.0030		1.20	1.08-1.34	0.0011		1.31	1.18-1.45	<0.0001
Prescriptions	1.02	1.01-1.04	0.0075		1.01	0.99-1.03	0.3969		1.02	1.00-1.03	0.0421
Hospitalisations	1.41	1.03-1.92	0.0295		1.34	1.09-1.66	0.0061		1.34	1.10-1.63	0.0043
Duration of diabetes (years)	1.02	0.98-1.07	0.3210		1.01	0.98-1.04	0.6083		1.04	1.01-1.07	0.0084
Complications	2.65	2.21-3.17	<0.0001		2.63	2.29-3.01	<0.0001		2.63	2.31-2.99	<0.0001
Glucose lowering therapies	1.38	1.05-1.80	0.0189		1.24	1.03-1.49	0.0229		1.10	0.92-1.32	0.3050
Insulin prescription	3.88	2.07-7.28	<0.0001		2.59	1.75-3.84	<0.0001		2.53	1.72-3.73	<0.0001

Study sizes across exposures after 1:1 propensity score matching are provided in Supplementary Table 8, as they are the same between univariate and multivariate analyses.

N/A indicates no observations for the covariate after propensity score matching.

Supplementary Table 10: Multivariate hazard ratios (with 95% CIs and p-values) for minor or major amputation risk by covariates across QOF exposures, *among those who do not meet either of the other two targets*, after 1:1 propensity score matching, including the adjusted study size (n) and C-statistic (also with 95% CI).

	Exposure Definition										
	Achieve HbA1c QOF Target				Achieve Blood Pressure QOF Target				Achieve Cholesterol QOF Target		
	Hazard Ratio	95% CI	p		Hazard Ratio	95% CI	p		Hazard Ratio	95% CI	p
Exposure	0.42	0.22-0.78	0.0064		0.79	0.53-1.17	0.2336		0.65	0.44-0.96	0.0316
Age	1.00	0.97-1.03	0.8735		1.00	0.98-1.02	0.6438		0.98	0.96-0.99	0.0120
Sex: Female	0.97	0.51-1.85	0.9238		1.03	0.65-1.63	0.9008		1.35	0.86-2.13	0.1935
Ethnicity: Asian	N/A	N/A	N/A		0.77	0.32-1.85	0.5567		0.34	0.10-1.10	0.0718
Ethnicity: Black	N/A	N/A	N/A		0.35	0.05-2.57	0.3033		1.39	0.50-3.86	0.5306
Ethnicity: Mixed	N/A	N/A	N/A		N/A	N/A	N/A		1.20	0.17-8.72	0.8564
Ethnicity: Other	1.03	0.13-7.99	0.9764		0.79	0.11-5.76	0.8126		0.68	0.09-4.98	0.7034
IMD	1.07	1.01-1.13	0.0188		1.01	0.97-1.04	0.7646		1.02	0.98-1.06	0.2550
North West	N/A	N/A	N/A		0.91	0.30-2.74	0.8712		1.15	0.26-4.99	0.8541
Yorkshire & Humber	N/A	N/A	N/A		0.77	0.17-3.51	0.7337		1.09	0.20-6.03	0.9206
East Midlands	N/A	N/A	N/A		1.03	0.23-4.70	0.9684		1.45	0.24-8.80	0.6840
West Midlands	N/A	N/A	N/A		0.72	0.22-2.34	0.5850		0.77	0.16-3.67	0.7387
East of England	N/A	N/A	N/A		0.59	0.17-2.01	0.3990		1.11	0.24-5.11	0.8938
South West	N/A	N/A	N/A		0.79	0.25-2.43	0.6781		1.35	0.31-5.93	0.6914
South Central	N/A	N/A	N/A		0.71	0.22-2.28	0.5622		1.00	0.22-4.57	0.9970
London	N/A	N/A	N/A		0.64	0.20-2.04	0.4559		1.24	0.28-5.47	0.7770
South East Coast	N/A	N/A	N/A		0.55	0.17-1.82	0.3280		1.14	0.25-5.14	0.8681
BMI: Underweight	N/A	N/A	N/A		1.49	0.19-11.57	0.7036		0.65	0.08-5.17	0.6833
BMI: Overweight	N/A	N/A	N/A		1.07	0.14-8.04	0.9449		0.51	0.07-3.90	0.5183
BMI: Obese	N/A	N/A	N/A		1.27	0.17-9.53	0.8137		0.71	0.09-5.30	0.7365
BMI: Missing	N/A	N/A	N/A		0.88	0.05-14.91	0.9300		1.19	0.10-13.74	0.8905
Ex-Smoker	0.91	0.47-1.77	0.7845		1.18	0.74-1.88	0.4876		0.86	0.53-1.39	0.5463
Current Smoker	1.55	0.72-3.32	0.2597		0.95	0.54-1.68	0.8719		1.68	1.04-2.72	0.0333
Smoking: Missing	N/A	N/A	N/A		N/A	N/A	N/A		N/A	N/A	N/A
Alcohol: 1-14	1.57	0.60-4.11	0.3612		1.63	0.86-3.10	0.1315		1.29	0.72-2.33	0.3970
Alcohol: 15-42	0.50	0.09-2.68	0.4169		2.34	0.99-5.51	0.0526		1.38	0.62-3.08	0.4309
Alcohol: >42	4.32	1.05-17.81	0.0430		3.82	1.17-12.43	0.0260		2.49	0.91-6.84	0.0766
Alcohol: Missing	2.76	0.90-8.46	0.0752		2.29	1.09-4.80	0.0291		1.87	0.94-3.74	0.0762
Morbidities	0.83	0.69-1.01	0.0653		0.84	0.73-0.96	0.0126		0.92	0.81-1.04	0.1947
Prescriptions	1.01	0.98-1.04	0.6691		0.98	0.95-1.01	0.1268		0.99	0.97-1.02	0.6184
Hospitalisations	0.85	0.58-1.25	0.4137		1.08	0.83-1.39	0.5750		0.96	0.74-1.25	0.7812
Duration of diabetes (years)	1.01	0.96-1.07	0.6581		0.99	0.96-1.03	0.7053		1.02	1.00-1.05	0.1049
Complications	2.83	2.26-3.54	<0.0001		2.91	2.47-3.43	<0.0001		2.95	2.51-3.46	<0.0001
Glucose lowering therapies	1.05	0.75-1.48	0.7760		1.10	0.86-1.40	0.4635		0.96	0.76-1.21	0.7316
Insulin prescription	1.32	0.63-2.78	0.4626		1.27	0.81-2.00	0.2984		1.04	0.67-1.63	0.8490
	Value	95% CI (lower)	95% CI (upper)		Value	95% CI (lower)	95% CI (upper)		Value	95% CI (lower)	95% CI (upper)
n after matching	8,362				8,326				9,938		
C-statistic	0.9057	0.9048	0.9067		0.8683	0.8677	0.8690		0.8710	0.8703	0.8717

Reference groups for categorical covariates include: white (ethnicity); North East (region); normal weight (BMI); non-smoker (smoking status); 0 units (alcohol consumption); and no insulin prescription (insulin use).

N/A indicates no observations for the covariate after propensity score matching.

Supplementary Figure 1: Kaplan-Meier survival curves (and corresponding 95% CIs) for minor or major amputation risk after 1:1 propensity score matching across exposure definitions.

Exposure: HbA1c QOF Target

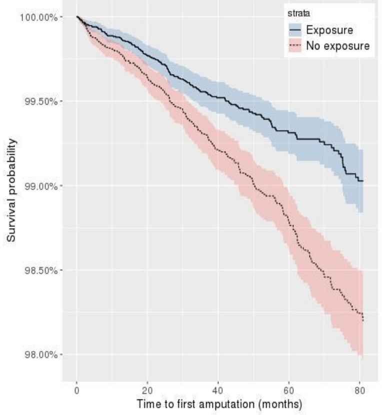

Exposure: BP QOF Target

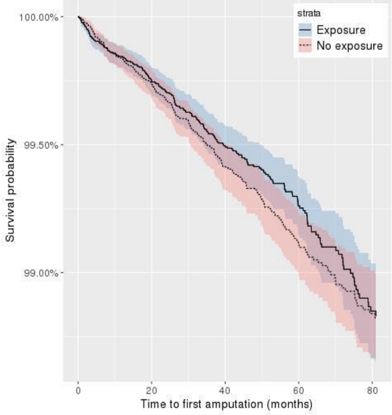

Exposure: Cholesterol QOF Target

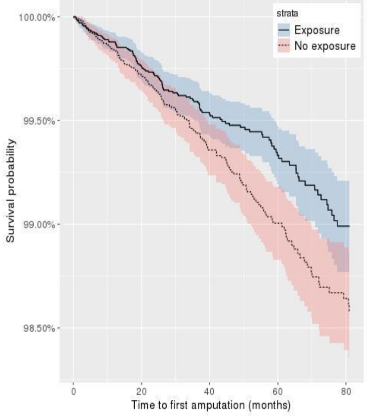

Exposure: 4-6 NDA Processes (vs. 0-3 NDA Processes)

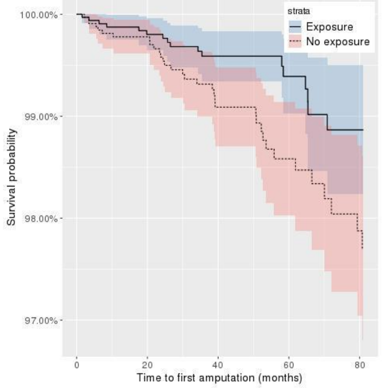

Exposure: 7-9 NDA Processes (vs. 0-3 NDA Processes)

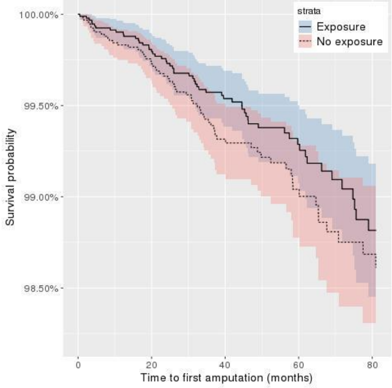

Exposure: 7-9 NDA Processes (vs. 4-6 NDA Processes)

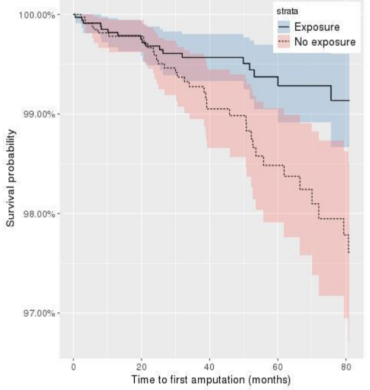

Exposure: All QOF Targets

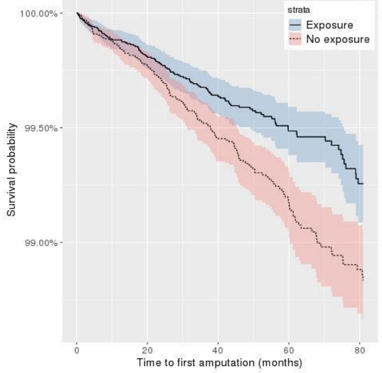

Exposure: All NDA Processes

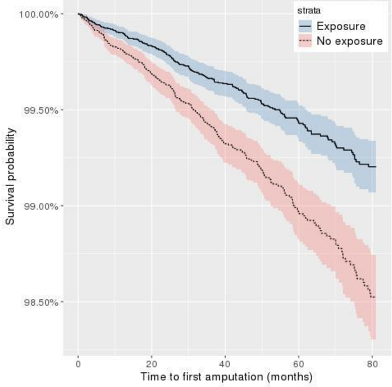

Exposure: All QOF & NDA Targets

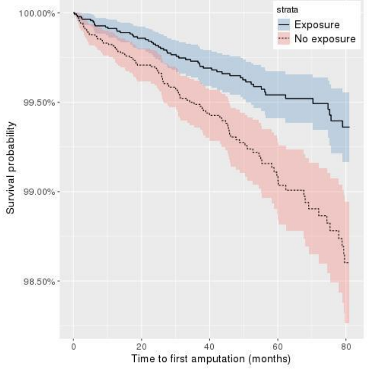

Supplementary Figure 2: Kaplan-Meier survival curves (and corresponding 95% CIs) for minor or major amputation risk after 1:1 propensity score matching across QOF exposure definitions, *among those who meet all other QOF targets*.

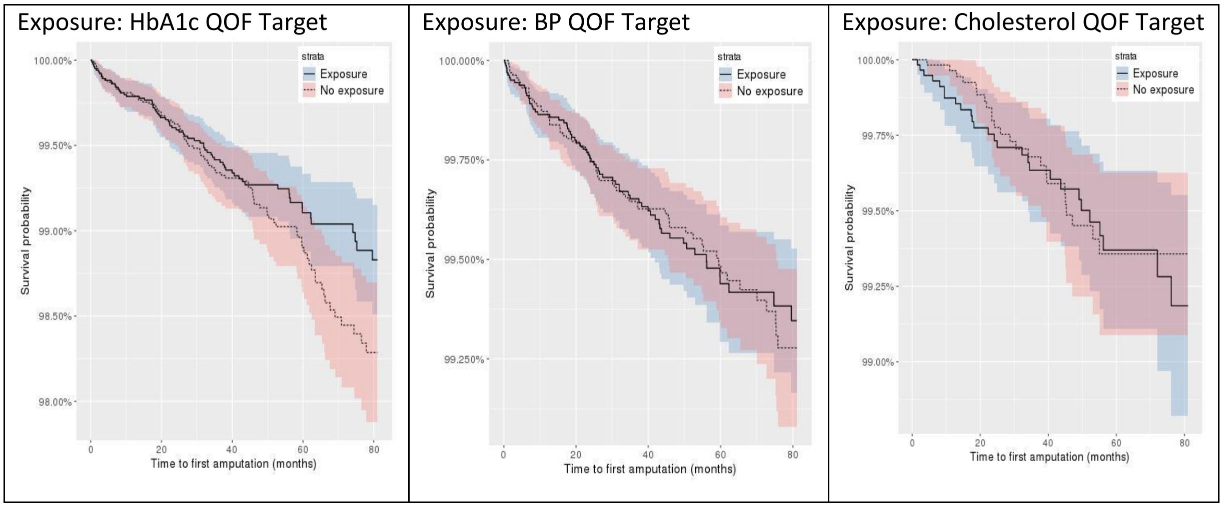

Supplementary Figure 3: Kaplan-Meier survival curves (and corresponding 95% CIs) for minor or major amputation risk after 1:1 propensity score matching across QOF exposure definitions, *among those who do not meet either of the other two QOF targets*.

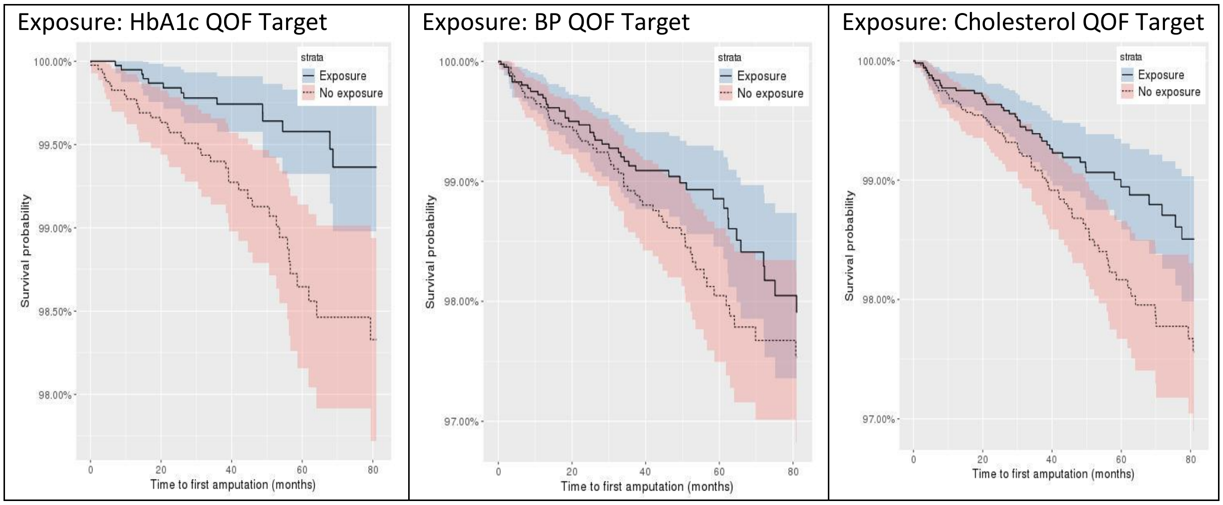

Supplementary Table 11: Univariate hazard ratios (with corresponding 95% CIs and p-values) for major-only amputation risk by each covariate across QOF exposure definitions after 1:1 propensity score matching.

	Exposure Definition										
	Achieve HbA1c QOF Target				Achieve Blood Pressure QOF Target				Achieve Cholesterol QOF Target		
	Hazard Ratio	95% CI	p		Hazard Ratio	95% CI	p		Hazard Ratio	95% CI	p
Exposure	0.65	0.47-0.91	0.0114		0.84	0.62-1.15	0.2777		0.86	0.58-1.27	0.4502
Age	1.03	1.01-1.04	0.0001		1.02	1.01-1.04	0.0009		1.03	1.01-1.05	0.0007
Sex: Female	0.78	0.55-1.09	0.1412		0.88	0.64-1.20	0.4239		0.90	0.61-1.32	0.5871
Ethnicity: Asian	0.42	0.17-1.02	0.0546		0.44	0.18-1.06	0.0676		0.43	0.14-1.35	0.1484
Ethnicity: Black	0.24	0.03-1.72	0.1555		0.46	0.11-1.87	0.2798		0.32	0.05-2.32	0.2610
Ethnicity: Mixed	0.87	0.12-6.24	0.8925		N/A	N/A	N/A		N/A	N/A	N/A
Ethnicity: Other	0.48	0.07-3.41	0.4609		N/A	N/A	N/A		N/A	N/A	N/A
IMD	1.01	0.98-1.04	0.4538		1.02	0.99-1.05	0.1256		1.01	0.98-1.05	0.4734
North West	1.34	0.91-1.99	0.1410		1.50	1.05-2.15	0.0267		1.36	0.85-2.16	0.1953
Yorkshire & Humber	0.53	0.17-1.68	0.2824		0.30	0.08-1.23	0.0949		N/A	N/A	N/A
East Midlands	0.95	0.30-2.99	0.9332		0.28	0.04-1.97	0.1997		1.34	0.42-4.22	0.6199
West Midlands	0.91	0.54-1.53	0.7151		0.93	0.57-1.52	0.7765		1.19	0.68-2.10	0.5390
East of England	0.85	0.48-1.51	0.5841		0.76	0.43-1.34	0.3466		0.76	0.37-1.57	0.4584
South West	1.22	0.78-1.90	0.3909		1.27	0.84-1.92	0.2595		1.64	1.02-2.65	0.0427
South Central	0.89	0.53-1.50	0.6736		0.69	0.40-1.20	0.1885		0.79	0.41-1.52	0.4843
London	0.75	0.44-1.28	0.2966		0.82	0.50-1.34	0.4258		0.63	0.32-1.25	0.1845
South East Coast	1.15	0.72-1.83	0.5530		1.35	0.89-2.04	0.1529		0.98	0.55-1.75	0.9411
BMI: Underweight	1.72	1.14-2.60	0.0101		1.74	1.19-2.55	0.0045		1.68	1.04-2.71	0.0343
BMI: Overweight	0.97	0.68-1.37	0.8512		0.88	0.63-1.23	0.4563		0.84	0.54-1.29	0.4151
BMI: Obese	0.75	0.54-1.04	0.0798		0.78	0.57-1.06	0.1178		0.84	0.57-1.23	0.3677
BMI: Missing	2.35	0.87-6.35	0.0918		2.28	0.84-6.14	0.1042		1.62	0.40-6.57	0.4996
Ex-Smoker	1.33	0.96-1.85	0.0910		1.27	0.93-1.73	0.1367		1.47	0.99-2.18	0.0532
Current Smoker	1.67	1.13-2.46	0.0097		1.97	1.37-2.83	0.0002		2.01	1.30-3.11	0.0016
Smoking: Missing	N/A	N/A	N/A		N/A	N/A	N/A		N/A	N/A	N/A
Alcohol: 1-14	0.76	0.55-1.05	0.0991		0.80	0.59-1.09	0.1564		0.63	0.43-0.93	0.0213
Alcohol: 15-42	0.50	0.24-1.08	0.0770		0.58	0.31-1.10	0.0960		0.58	0.25-1.31	0.1886
Alcohol: >42	1.73	0.71-4.22	0.2287		1.38	0.57-3.36	0.4774		1.59	0.59-4.32	0.3631
Alcohol: Missing	1.30	0.85-1.98	0.2277		1.34	0.89-2.03	0.1621		1.50	0.92-2.44	0.1044
Morbidities	1.38	1.27-1.50	<0.0001		1.44	1.33-1.55	<0.0001		1.43	1.29-1.58	<0.0001
Prescriptions	1.01	1.00-1.03	0.0787		1.02	1.01-1.03	0.0007		1.02	1.00-1.03	0.0149
Hospitalisations	1.64	1.43-1.88	<0.0001		1.65	1.46-1.86	<0.0001		1.58	1.34-1.87	<0.0001
Duration of diabetes (years)	1.02	1.00-1.05	0.0800		1.01	0.99-1.04	0.3318		1.02	0.99-1.05	0.2501
Complications	2.55	2.28-2.85	<0.0001		2.84	2.55-3.17	<0.0001		2.78	2.43-3.18	<0.0001
Glucose lowering therapies	1.01	0.86-1.20	0.8790		1.18	1.02-1.36	0.0306		1.27	1.06-1.52	0.0111
Insulin prescription	3.46	2.48-4.81	<0.0001		4.31	3.15-5.90	<0.0001		3.69	2.46-5.54	<0.0001

Study sizes across exposures after 1:1 propensity score matching are provided in Supplementary Table 14, as they are the same between univariate and multivariate analyses.

Supplementary Table 12: Univariate hazard ratios (with corresponding 95% CIs and p-values) for major-only amputation risk by each covariate across NDA exposure definitions after 1:1 propensity score matching.

	**Exposure Definition**								
	**Meet 4-6 NDA Processes (vs. Meet 0-3 NDA Processes)**			**Meet 7-9 NDA Processes (vs. Meet 0-3 NDA Processes)**			**Meet 7-9 NDA Processes (vs. Meet 4-6 NDA Processes)**		
	**Hazard Ratio**	**95% CI**	**p**	**Hazard Ratio**	**95% CI**	**p**	**Hazard Ratio**	**95% CI**	**p**
Exposure	0.78	0.37-1.62	0.5046	0.36	0.19-0.69	0.0021	0.12	0.03-0.53	0.0049
Age	1.01	0.99-1.04	0.3531	1.01	0.99-1.04	0.2725	1.02	0.99-1.06	0.2283
Sex: Female	0.88	0.42-1.84	0.7319	0.49	0.26-0.93	0.0304	1.50	0.58-3.89	0.4026
Ethnicity: Asian	0.38	0.05-2.81	0.3446	0.77	0.24-2.48	0.6604	0.75	0.10-5.62	0.7755
Ethnicity: Black	N/A	N/A	N/A	N/A	N/A	N/A	N/A	N/A	N/A
Ethnicity: Mixed	N/A	N/A	N/A	N/A	N/A	N/A	N/A	N/A	N/A
Ethnicity: Other	N/A	N/A	N/A	N/A	N/A	N/A	N/A	N/A	N/A
IMD	1.01	0.94-1.07	0.8369	1.00	0.95-1.05	0.9219	0.96	0.88-1.05	0.3936
North West	1.58	0.68-3.71	0.2889	1.70	0.88-3.30	0.1143	1.51	0.49-4.64	0.4681
Yorkshire & Humber	1.00	0.14-7.36	0.9989	0.55	0.08-4.03	0.5603	N/A	N/A	N/A
East Midlands	1.75	0.24-12.88	0.5824	N/A	N/A	N/A	2.34	0.31-17.66	0.4092
West Midlands	0.87	0.26-2.88	0.8233	0.93	0.37-2.36	0.8803	1.54	0.44-5.35	0.4986
East of England	1.42	0.50-4.09	0.5111	1.07	0.42-2.71	0.8878	1.11	0.25-4.86	0.8879
South West	0.74	0.22-2.46	0.6267	1.76	0.87-3.56	0.1142	1.40	0.40-4.87	0.5974
South Central	0.51	0.12-2.16	0.3639	0.94	0.37-2.37	0.8894	0.45	0.06-3.37	0.4339
London	0.98	0.34-2.82	0.9722	0.47	0.15-1.53	0.2126	0.41	0.06-3.13	0.3932
South East Coast	1.04	0.36-2.99	0.9426	0.48	0.15-1.56	0.2249	0.87	0.20-3.80	0.8528
BMI: Underweight	0.46	0.11-1.92	0.2836	0.79	0.31-2.01	0.6246	0.78	0.18-3.40	0.7366
BMI: Overweight	0.74	0.32-1.74	0.4966	1.26	0.69-2.31	0.4481	0.30	0.07-1.30	0.1076
BMI: Obese	1.71	0.79-3.68	0.1703	0.84	0.47-1.50	0.5498	3.19	1.04-9.79	0.0424
BMI: Missing	1.35	0.18-9.90	0.7698	2.28	0.55-9.43	0.2534	N/A	N/A	N/A
Ex-Smoker	1.67	0.80-3.49	0.1756	1.35	0.75-2.43	0.3247	1.50	0.57-3.94	0.4111
Current Smoker	2.16	1.00-4.64	0.0493	1.33	0.62-2.86	0.4643	2.87	1.09-7.53	0.0327
Smoking: Missing	N/A	N/A	N/A	N/A	N/A	N/A	N/A	N/A	N/A
Alcohol: 1-14	0.92	0.44-1.90	0.8204	0.98	0.54-1.76	0.9409	0.50	0.19-1.36	0.1772
Alcohol: 15-42	0.30	0.04-2.21	0.2385	0.45	0.11-1.84	0.2651	N/A	N/A	N/A
Alcohol: >42	1.14	0.16-8.41	0.8945	1.70	0.41-7.02	0.4626	2.08	0.28-15.72	0.4761
Alcohol: Missing	1.65	0.73-3.73	0.2268	1.14	0.53-2.46	0.7314	1.77	0.62-5.03	0.2827
Morbidities	1.14	0.91-1.41	0.2524	1.28	1.09-1.49	0.0022	1.00	0.74-1.36	0.9978
Prescriptions	1.01	0.98-1.04	0.5035	1.01	0.99-1.04	0.3209	1.00	0.95-1.06	0.8978
Hospitalisations	1.51	1.11-2.05	0.0089	1.56	1.29-1.88	<0.0001	1.26	0.79-1.99	0.3359
Duration of diabetes (years)	0.99	0.92-1.06	0.7387	1.00	0.94-1.05	0.8878	0.98	0.89-1.09	0.7216
Complications	2.75	2.15-3.53	<0.0001	2.66	2.17-3.27	<0.0001	2.40	1.74-3.31	<0.0001
Glucose lowering therapies	1.17	0.83-1.66	0.3747	1.31	1.00-1.72	0.0533	1.06	0.69-1.63	0.7983
Insulin prescription	3.92	1.78-8.61	0.0007	4.73	2.63-8.51	<0.0001	2.41	0.79-7.39	0.1241

Study sizes across exposures after 1:1 propensity score matching are provided in Supplementary Table 15, as they are the same between univariate and multivariate analyses.

N/A indicates no observations for the covariate after propensity score matching.

Supplementary Table 13: Univariate hazard ratios (with corresponding 95% CIs and p-values) for major-only amputation risk by each covariate across NDA and QOF exposure definitions after 1:1 propensity score matching.

	Exposure Definition								
	Achieve All QOF Targets			Meet All NDA Processes			Achieve All QOF & NDA Targets		
	Hazard Ratio	95% CI	p	Hazard Ratio	95% CI	p	Hazard Ratio	95% CI	p
Exposure	0.76	0.52-1.10	0.1449	0.52	0.38-0.71	<0.0001	0.75	0.44-1.28	0.2910
Age	1.01	1.00-1.03	0.1101	1.02	1.00-1.03	0.0133	1.03	1.00-1.05	0.0383
Sex: Female	0.68	0.46-1.01	0.0558	0.71	0.52-0.98	0.0364	0.60	0.33-1.08	0.0873
Ethnicity: Asian	0.57	0.21-1.54	0.2678	0.38	0.14-1.02	0.0555	0.34	0.05-2.47	0.2869
Ethnicity: Black	N/A	N/A	N/A	0.63	0.16-2.56	0.5229	N/A	N/A	N/A
Ethnicity: Mixed	N/A	N/A	N/A	0.76	0.11-5.43	0.7852	N/A	N/A	N/A
Ethnicity: Other	N/A	N/A	N/A	0.48	0.07-3.46	0.4702	1.66	0.23-12.00	0.6164
IMD	1.01	0.98-1.05	0.4241	1.02	0.99-1.05	0.1541	1.03	0.98-1.08	0.2553
North West	1.41	0.91-2.19	0.1266	1.31	0.91-1.90	0.1485	2.01	1.12-3.62	0.0195
Yorkshire & Humber	0.22	0.03-1.56	0.1299	0.47	0.15-1.46	0.1894	0.48	0.07-3.45	0.4639
East Midlands	1.22	0.39-3.84	0.7338	0.53	0.13-2.13	0.3712	N/A	N/A	N/A
West Midlands	1.06	0.61-1.86	0.8342	0.97	0.60-1.57	0.9056	1.50	0.73-3.06	0.2706
East of England	0.67	0.33-1.38	0.2766	0.85	0.50-1.45	0.5542	0.68	0.25-1.88	0.4587
South West	1.61	1.01-2.56	0.0462	1.36	0.91-2.03	0.1331	0.65	0.26-1.64	0.3644
South Central	0.57	0.28-1.17	0.1241	0.85	0.52-1.41	0.5289	0.29	0.07-1.20	0.0878
London	0.80	0.44-1.46	0.4642	0.71	0.42-1.18	0.1883	0.84	0.36-1.96	0.6824
South East Coast	1.18	0.70-2.01	0.5358	1.27	0.83-1.93	0.2674	1.48	0.72-3.03	0.2831
BMI: Underweight	1.79	1.17-2.75	0.0078	1.45	0.98-2.16	0.0639	1.75	0.94-3.27	0.0801
BMI: Overweight	0.83	0.56-1.24	0.3734	0.84	0.60-1.17	0.3044	1.12	0.65-1.95	0.6795
BMI: Obese	0.83	0.57-1.21	0.3248	0.92	0.68-1.25	0.6119	0.60	0.34-1.06	0.0801
BMI: Missing	N/A	N/A	N/A	2.23	0.71-6.97	0.1698	N/A	N/A	N/A
Ex-Smoker	1.23	0.85-1.79	0.2725	1.37	1.01-1.86	0.0435	1.34	0.78-2.30	0.2830
Current Smoker	2.25	1.46-3.47	0.0002	1.75	1.20-2.54	0.0034	1.24	0.59-2.64	0.5692
Smoking: Missing	N/A	N/A	N/A	N/A	N/A	N/A	N/A	N/A	N/A
Alcohol: 1-14	0.84	0.58-1.23	0.3719	0.78	0.58-1.07	0.1213	0.82	0.48-1.42	0.4849
Alcohol: 15-42	0.81	0.41-1.60	0.5438	0.70	0.39-1.25	0.2279	0.70	0.25-1.95	0.4975
Alcohol: >42	0.95	0.23-3.85	0.9440	1.25	0.46-3.37	0.6577	N/A	N/A	N/A
Alcohol: Missing	1.29	0.76-2.19	0.3455	1.49	0.97-2.28	0.0666	2.20	1.10-4.37	0.0252
Morbidities	1.39	1.27-1.53	<0.0001	1.39	1.28-1.50	<0.0001	1.38	1.20-1.58	<0.0001
Prescriptions	1.02	1.01-1.04	0.0002	1.02	1.01-1.03	0.0002	1.03	1.00-1.05	0.0178
Hospitalisations	1.74	1.51-2.01	<0.0001	1.62	1.40-1.88	<0.0001	1.94	1.45-2.59	<0.0001
Duration of diabetes (years)	1.01	0.98-1.05	0.3791	1.02	0.99-1.04	0.2071	1.03	0.99-1.07	0.1254
Complications	2.50	2.19-2.84	<0.0001	2.74	2.46-3.06	<0.0001	2.53	2.09-3.06	<0.0001
Glucose lowering therapies	1.30	1.07-1.56	0.0066	1.24	1.07-1.43	0.0041	1.33	1.01-1.74	0.0386
Insulin prescription	5.17	3.45-7.73	<0.0001	4.69	3.44-6.41	<0.0001	4.72	2.56-8.69	<0.0001

Study sizes across exposures after 1:1 propensity score matching are provided in Supplementary Table 16, as they are the same between univariate and multivariate analyses.

N/A indicates no observations for the covariate after propensity score matching.

Supplementary Table 14: Multivariate hazard ratios (with corresponding 95% CIs and p-values) for major-only amputation risk by each covariate across QOF exposure definitions after 1:1 propensity score matching, including the adjusted study size (n) and C-statistic (also with corresponding 95% CI).

	**Exposure Definition**								
	**Achieve HbA1c QOF Target**			**Achieve Blood Pressure QOF Target**			**Achieve Cholesterol QOF Target**		
	**Hazard Ratio**	**95% CI**	**p**	**Hazard Ratio**	**95% CI**	**p**	**Hazard Ratio**	**95% CI**	**p**
Exposure	0.69	0.49-0.97	0.0302	0.81	0.59-1.10	0.1754	0.84	0.56-1.24	0.3739
Age	0.99	0.98-1.01	0.4852	0.99	0.97-1.01	0.1735	1.00	0.98-1.02	0.9465
Sex: Female	1.19	0.82-1.73	0.3605	1.51	1.07-2.13	0.0203	1.50	0.97-2.33	0.0673
Ethnicity: Asian	0.37	0.15-0.93	0.0339	0.41	0.16-1.02	0.0554	0.41	0.12-1.34	0.1389
Ethnicity: Black	0.26	0.04-1.90	0.1852	0.52	0.13-2.11	0.3567	0.37	0.05-2.67	0.3219
Ethnicity: Mixed	0.85	0.12-6.15	0.8754	N/A	N/A	N/A	N/A	N/A	N/A
Ethnicity: Other	0.56	0.08-4.02	0.5615	N/A	N/A	N/A	N/A	N/A	N/A
IMD	1.01	0.98-1.04	0.5894	1.02	0.99-1.05	0.2976	1.01	0.97-1.05	0.6243
North West	1.47	0.45-4.82	0.5214	1.99	0.61-6.46	0.2531	4.10	0.55-30.59	0.1688
Yorkshire & Humber	0.60	0.12-2.98	0.5315	0.44	0.07-2.65	0.3689	N/A	N/A	N/A
East Midlands	1.07	0.21-5.30	0.9378	0.42	0.04-4.06	0.4539	4.18	0.43-40.60	0.2178
West Midlands	1.03	0.30-3.56	0.9591	1.48	0.43-5.08	0.5293	4.02	0.52-30.96	0.1810
East of England	1.01	0.29-3.57	0.9890	1.16	0.32-4.12	0.8226	2.52	0.31-20.54	0.3869
South West	1.28	0.38-4.30	0.6861	1.76	0.53-5.85	0.3588	4.49	0.60-33.79	0.1445
South Central	1.11	0.32-3.86	0.8678	1.16	0.33-4.11	0.8159	2.73	0.34-21.75	0.3435
London	0.86	0.25-2.98	0.8120	1.19	0.35-4.08	0.7774	1.96	0.25-15.64	0.5249
South East Coast	1.28	0.38-4.34	0.6900	1.88	0.56-6.28	0.3079	2.98	0.38-23.27	0.2971
BMI: Underweight	N/A	N/A	N/A	1.19	0.16-8.77	0.8620	0.87	0.12-6.56	0.8956
BMI: Overweight	N/A	N/A	N/A	0.69	0.10-5.05	0.7183	0.48	0.06-3.55	0.4701
BMI: Obese	N/A	N/A	N/A	0.61	0.08-4.46	0.6301	0.50	0.07-3.66	0.4922
BMI: Missing	N/A	N/A	N/A	1.48	0.16-13.31	0.7287	0.80	0.07-8.92	0.8534
Ex-Smoker	1.25	0.85-1.85	0.2640	1.29	0.89-1.87	0.1865	1.69	1.04-2.75	0.0355
Current Smoker	1.90	1.19-3.03	0.0072	2.11	1.36-3.27	0.0009	2.70	1.56-4.68	0.0004
Smoking: Missing	N/A	N/A	N/A	N/A	N/A	N/A	N/A	N/A	N/A
Alcohol: 1-14	0.68	0.45-1.03	0.0678	0.78	0.52-1.17	0.2317	0.58	0.35-0.95	0.0301
Alcohol: 15-42	0.44	0.19-1.02	0.0563	0.56	0.27-1.16	0.1164	0.47	0.19-1.17	0.1049
Alcohol: >42	1.07	0.41-2.81	0.8911	0.90	0.34-2.35	0.8256	0.95	0.32-2.84	0.9287
Alcohol: Missing	1.33	0.79-2.24	0.2802	1.29	0.77-2.15	0.3272	1.32	0.73-2.40	0.3575
Morbidities	0.91	0.82-1.01	0.0839	0.92	0.83-1.02	0.1045	0.91	0.81-1.03	0.1531
Prescriptions	0.99	0.97-1.01	0.3609	0.99	0.97-1.01	0.3442	0.99	0.96-1.01	0.3284
Hospitalisations	1.27	1.06-1.51	0.0087	1.21	1.03-1.42	0.0225	1.17	0.94-1.45	0.1566
Duration of diabetes (years)	1.01	0.98-1.03	0.6315	0.99	0.96-1.02	0.4785	1.00	0.96-1.03	0.9406
Complications	2.55	2.23-2.92	<0.0001	2.86	2.51-3.26	<0.0001	2.82	2.40-3.32	<0.0001
Glucose lowering therapies	0.91	0.74-1.12	0.3860	0.99	0.81-1.20	0.8942	1.10	0.86-1.40	0.4593
Insulin prescription	1.85	1.29-2.66	0.0009	2.09	1.45-3.01	0.0001	1.52	0.94-2.45	0.0851
	**Value**	**95% CI (lower)**	**95% CI (upper)**	**Value**	**95% CI (lower)**	**95% CI (upper)**	**Value**	**95% CI (lower)**	**95% CI (upper)**
n after matching	52,838			69,540			40,930		
C-statistic	0.8585	0.8580	0.8591	0.8849	0.8845	0.8853	0.8958	0.8952	0.8964

Reference groups for categorical covariates include: white (ethnicity); North East (region); normal weight (BMI); non-smoker (smoking status); 0 units (alcohol consumption); and no insulin prescription (insulin use).

Supplementary Table 15: Multivariate hazard ratios (with corresponding 95% CIs and p-values) for major-only amputation risk by each covariate across NDA exposure definitions after 1:1 propensity score matching, including the adjusted study size (n) and C-statistic (also with corresponding 95% CI).

	Exposure Definition								
	Meet 4-6 NDA Processes (vs. Meet 0-3 NDA Processes)			Meet 7-9 NDA Processes (vs. Meet 0-3 NDA Processes)			Meet 7-9 NDA Processes (vs. Meet 4-6 NDA Processes)		
	Hazard Ratio	95% CI	p	Hazard Ratio	95% CI	p	Hazard Ratio	95% CI	p
Exposure	0.92	0.42-2.02	0.8356	0.32	0.16-0.61	0.0006	0.10	0.02-0.49	0.0042
Age	1.00	0.96-1.03	0.8624	0.99	0.96-1.02	0.6158	1.05	1.00-1.10	0.0483
Sex: Female	1.93	0.81-4.61	0.1365	0.74	0.36-1.52	0.4154	2.17	0.68-6.93	0.1899
Ethnicity: Asian	0.61	0.07-4.94	0.6401	0.63	0.18-2.20	0.4679	1.94	0.22-17.26	0.5539
Ethnicity: Black	N/A	N/A	N/A	N/A	N/A	N/A	N/A	N/A	N/A
Ethnicity: Mixed	N/A	N/A	N/A	N/A	N/A	N/A	N/A	N/A	N/A
Ethnicity: Other	N/A	N/A	N/A	N/A	N/A	N/A	N/A	N/A	N/A
IMD	1.00	0.93-1.07	0.9700	0.99	0.94-1.05	0.7886	0.94	0.85-1.03	0.1957
North West	N/A	N/A	N/A	1.68	0.22-13.05	0.6212	N/A	N/A	N/A
Yorkshire & Humber	N/A	N/A	N/A	0.54	0.03-8.65	0.6599	N/A	N/A	N/A
East Midlands	N/A	N/A	N/A	N/A	N/A	N/A	N/A	N/A	N/A
West Midlands	N/A	N/A	N/A	0.97	0.11-8.40	0.9768	N/A	N/A	N/A
East of England	N/A	N/A	N/A	1.12	0.13-9.79	0.9199	N/A	N/A	N/A
South West	N/A	N/A	N/A	1.60	0.20-12.72	0.6551	N/A	N/A	N/A
South Central	N/A	N/A	N/A	0.90	0.10-7.92	0.9213	N/A	N/A	N/A
London	N/A	N/A	N/A	0.56	0.06-5.43	0.6162	N/A	N/A	N/A
South East Coast	N/A	N/A	N/A	0.51	0.05-5.05	0.5679	N/A	N/A	N/A
BMI: Underweight	N/A	N/A	N/A	N/A	N/A	N/A	N/A	N/A	N/A
BMI: Overweight	N/A	N/A	N/A	N/A	N/A	N/A	N/A	N/A	N/A
BMI: Obese	N/A	N/A	N/A	N/A	N/A	N/A	N/A	N/A	N/A
BMI: Missing	N/A	N/A	N/A	N/A	N/A	N/A	N/A	N/A	N/A
Ex-Smoker	2.32	0.86-6.23	0.0963	1.21	0.61-2.42	0.5846	3.29	0.76-14.27	0.1115
Current Smoker	4.13	1.47-11.64	0.0073	1.39	0.57-3.39	0.4683	8.08	1.75-37.23	0.0074
Smoking: Missing	N/A	N/A	N/A	N/A	N/A	N/A	N/A	N/A	N/A
Alcohol: 1-14	1.10	0.37-3.28	0.8578	0.91	0.40-2.06	0.8257	0.42	0.11-1.65	0.2162
Alcohol: 15-42	0.43	0.05-4.01	0.4619	0.45	0.09-2.21	0.3285	N/A	N/A	N/A
Alcohol: >42	1.70	0.16-18.33	0.6607	0.99	0.20-4.97	0.9871	1.31	0.11-15.28	0.8320
Alcohol: Missing	2.22	0.69-7.18	0.1807	1.38	0.51-3.73	0.5295	1.20	0.31-4.62	0.7898
Morbidities	0.69	0.52-0.91	0.0079	0.86	0.70-1.05	0.1436	0.61	0.40-0.92	0.0179
Prescriptions	0.97	0.92-1.02	0.2048	0.97	0.92-1.01	0.1755	0.97	0.90-1.04	0.3606
Hospitalisations	1.45	0.96-2.19	0.0785	1.31	1.02-1.67	0.0358	1.54	0.95-2.51	0.0804
Duration of diabetes (years)	0.97	0.90-1.05	0.4717	0.98	0.92-1.04	0.4584	0.95	0.84-1.06	0.3534
Complications	3.62	2.62-5.01	<0.0001	2.72	2.11-3.49	<0.0001	3.26	2.12-5.02	<0.0001
Glucose lowering therapies	0.98	0.62-1.55	0.9273	1.16	0.81-1.65	0.4276	1.18	0.60-2.33	0.6287
Insulin prescription	2.06	0.82-5.16	0.1235	2.63	1.32-5.24	0.0061	1.28	0.34-4.80	0.7177
	Value	95% CI (lower)	95% CI (upper)	Value	95% CI (lower)	95% CI (upper)	Value	95% CI (lower)	95% CI (upper)
n after matching	6,774			18,960			6,820		
C-statistic	0.9152	0.9127	0.9177	0.8934	0.8921	0.8947	0.9499	0.9488	0.9510

Reference groups for categorical covariates include: white (ethnicity); North East (region); normal weight (BMI); non-smoker (smoking status); 0 units (alcohol consumption); and no insulin prescription (insulin use).
N/A indicates no observations for the covariate after propensity score matching.

Supplementary Table 16: Multivariate hazard ratios (with corresponding 95% CIs and p-values) for major-only amputation risk by each covariate across QOF and NDA exposure definitions after 1:1 propensity score matching, including the adjusted study size (n) and C-statistic (also with corresponding 95% CI).

	**Exposure Definition**								
	**Achieve All QOF Targets**			**Meet All NDA Processes**			**Achieve All QOF & NDA Targets**		
	**Hazard Ratio**	**95% CI**	**p**	**Hazard Ratio**	**95% CI**	**p**	**Hazard Ratio**	**95% CI**	**p**
Exposure	0.76	0.52-1.11	0.1595	0.54	0.39-0.74	0.0001	0.76	0.44-1.32	0.3327
Age	0.99	0.97-1.01	0.1716	0.99	0.97-1.01	0.1871	0.99	0.96-1.02	0.5872
Sex: Female	1.13	0.74-1.75	0.5680	1.26	0.88-1.79	0.2040	1.00	0.52-1.90	0.9911
Ethnicity: Asian	0.48	0.17-1.37	0.1718	0.33	0.12-0.91	0.0322	0.19	0.02-1.42	0.1053
Ethnicity: Black	N/A	N/A	N/A	0.66	0.16-2.72	0.5687	N/A	N/A	N/A
Ethnicity: Mixed	N/A	N/A	N/A	0.73	0.10-5.28	0.7581	N/A	N/A	N/A
Ethnicity: Other	N/A	N/A	N/A	0.54	0.07-3.86	0.5366	1.85	0.24-13.95	0.5528
IMD	1.01	0.97-1.04	0.6420	1.02	0.99-1.05	0.1641	1.02	0.97-1.07	0.4889
North West	3.54	0.48-26.15	0.2146	2.66	0.64-11.05	0.1790	3.09	0.39-24.25	0.2824
Yorkshire & Humber	0.61	0.04-9.74	0.7246	0.99	0.17-5.97	0.9942	0.95	0.06-15.79	0.9738
East Midlands	3.36	0.35-32.43	0.2956	1.09	0.15-7.80	0.9278	N/A	N/A	N/A
West Midlands	2.86	0.37-21.85	0.3107	1.97	0.46-8.48	0.3633	2.73	0.33-22.57	0.3504
East of England	1.93	0.24-15.58	0.5359	1.87	0.42-8.26	0.4069	1.42	0.15-13.40	0.7575
South West	3.98	0.53-29.66	0.1782	2.64	0.63-11.14	0.1851	1.30	0.15-11.63	0.8146
South Central	1.76	0.22-14.18	0.5971	1.96	0.45-8.55	0.3724	0.65	0.06-7.53	0.7295
London	2.28	0.30-17.54	0.4299	1.53	0.35-6.67	0.5717	1.57	0.18-13.52	0.6830
South East Coast	3.28	0.43-24.98	0.2510	2.60	0.61-11.05	0.1961	2.60	0.31-21.58	0.3759
BMI: Underweight	1.09	0.15-8.02	0.9353	1.23	0.17-9.06	0.8405	0.47	0.06-3.67	0.4734
BMI: Overweight	0.57	0.08-4.17	0.5779	0.78	0.11-5.68	0.8061	0.32	0.04-2.44	0.2726
BMI: Obese	0.54	0.07-3.99	0.5482	0.78	0.11-5.65	0.8044	0.20	0.03-1.55	0.1233
BMI: Missing	N/A	N/A	N/A	1.62	0.17-15.66	0.6792	N/A	N/A	N/A
Ex-Smoker	1.29	0.82-2.04	0.2774	1.32	0.92-1.90	0.1368	1.06	0.57-1.98	0.8534
Current Smoker	2.27	1.33-3.90	0.0028	1.88	1.20-2.96	0.0061	1.31	0.56-3.07	0.5383
Smoking: Missing	N/A	N/A	N/A	N/A	N/A	N/A	N/A	N/A	N/A
Alcohol: 1-14	0.86	0.52-1.41	0.5445	0.79	0.52-1.19	0.2574	0.98	0.44-2.15	0.9555
Alcohol: 15-42	0.80	0.36-1.79	0.5906	0.67	0.34-1.33	0.2509	0.74	0.22-2.55	0.6375
Alcohol: >42	0.86	0.20-3.75	0.8462	0.98	0.34-2.82	0.9699	N/A	N/A	N/A
Alcohol: Missing	1.45	0.75-2.80	0.2660	1.55	0.91-2.62	0.1054	2.65	1.03-6.81	0.0437
Morbidities	0.96	0.85-1.09	0.5254	0.91	0.82-1.01	0.0712	0.94	0.78-1.14	0.5489
Prescriptions	1.00	0.98-1.02	0.9218	0.99	0.97-1.01	0.4364	1.00	0.96-1.03	0.8536
Hospitalisations	1.35	1.12-1.61	0.0012	1.21	0.98-1.49	0.0709	1.34	0.95-1.89	0.0947
Duration of diabetes (years)	1.00	0.96-1.03	0.8502	1.00	0.97-1.02	0.7456	1.02	0.97-1.06	0.4439
Complications	2.35	2.01-2.75	<0.0001	2.76	2.42-3.14	<0.0001	2.46	1.93-3.13	<0.0001
Glucose lowering therapies	1.03	0.82-1.31	0.7826	1.00	0.83-1.22	0.9627	1.11	0.79-1.57	0.5437
Insulin prescription	2.41	1.52-3.82	0.0002	2.25	1.57-3.22	<0.0001	2.32	1.16-4.63	0.0171
	**Value**	**95% CI (lower)**	**95% CI (upper)**	**Value**	**95% CI (lower)**	**95% CI (upper)**	**Value**	**95% CI (lower)**	**95% CI (upper)**
n after matching	55,076			70,582			28,034		
C-statistic	0.8653	0.8647	0.8660	0.8709	0.8704	0.8713	0.8453	0.8435	0.8471

Reference groups for categorical covariates include: white (ethnicity); North East (region); normal weight (BMI); non-smoker (smoking status); 0 units (alcohol consumption); and no insulin prescription (insulin use).

N/A indicates no observations for the covariate after propensity score matching.

Supplementary Table 17: Univariate hazard ratios (with corresponding 95% CIs and p-values) for major-only amputation risk by each covariate across QOF exposure definitions, *among those who meet all other QOF targets*, after 1:1 propensity score matching.

	**Exposure Definition**										
	**Achieve HbA1c QOF Target**				**Achieve Blood Pressure QOF Target**				**Achieve Cholesterol QOF Target**		
	**Hazard Ratio**	**95% CI**	**p**		**Hazard Ratio**	**95% CI**	**p**		**Hazard Ratio**	**95% CI**	**p**
Exposure	1.01	0.62-1.65	0.9742		1.05	0.61-1.81	0.8650		0.54	0.18-1.60	0.2630
Age	1.03	1.00-1.05	0.0174		1.06	1.03-1.09	<0.0001		1.03	0.98-1.08	0.2646
Sex: Female	0.63	0.37-1.07	0.0878		0.73	0.41-1.29	0.2818		0.61	0.21-1.77	0.3678
Ethnicity: Asian	0.16	0.02-1.17	0.0715		0.60	0.15-2.47	0.4793		N/A	N/A	N/A
Ethnicity: Black	0.70	0.10-5.04	0.7217		N/A	N/A	N/A		N/A	N/A	N/A
Ethnicity: Mixed	2.03	0.28-14.67	0.4813		N/A	N/A	N/A		N/A	N/A	N/A
Ethnicity: Other	1.18	0.16-8.51	0.8700		N/A	N/A	N/A		N/A	N/A	N/A
IMD	0.99	0.95-1.04	0.8027		0.98	0.93-1.03	0.4421		0.96	0.87-1.05	0.3532
North West	0.78	0.38-1.57	0.4848		1.72	0.93-3.17	0.0844		1.23	0.34-4.40	0.7539
Yorkshire & Humber	1.23	0.39-3.94	0.7220		0.47	0.07-3.41	0.4562		N/A	N/A	N/A
East Midlands	0.68	0.09-4.91	0.7028		N/A	N/A	N/A		N/A	N/A	N/A
West Midlands	0.64	0.26-1.59	0.3346		0.81	0.32-2.03	0.6510		1.19	0.27-5.33	0.8178
East of England	0.72	0.29-1.79	0.4800		0.73	0.26-2.02	0.5418		0.69	0.09-5.27	0.7193
South West	1.82	1.01-3.31	0.0471		0.97	0.44-2.15	0.9377		1.80	0.50-6.45	0.3677
South Central	1.05	0.50-2.20	0.9035		0.45	0.14-1.45	0.1827		1.26	0.28-5.63	0.7624
London	0.71	0.31-1.65	0.4313		0.56	0.20-1.55	0.2625		1.07	0.24-4.77	0.9311
South East Coast	1.48	0.77-2.83	0.2385		2.25	1.20-4.22	0.0113		0.54	0.07-4.12	0.5510
BMI: Underweight	1.50	0.80-2.82	0.2059		2.67	1.48-4.81	0.0011		1.29	0.36-4.61	0.6992
BMI: Overweight	0.93	0.55-1.58	0.7949		1.29	0.74-2.25	0.3640		0.75	0.23-2.39	0.6245
BMI: Obese	0.71	0.43-1.17	0.1743		0.37	0.20-0.69	0.0016		1.17	0.41-3.33	0.7724
BMI: Missing	4.92	1.54-15.69	0.0071		N/A	N/A	N/A		N/A	N/A	N/A
Ex-Smoker	1.20	0.73-1.97	0.4826		2.49	1.42-4.35	0.0014		1.36	0.47-3.93	0.5669
Current Smoker	1.69	0.92-3.12	0.0910		1.27	0.60-2.71	0.5276		2.47	0.77-7.86	0.1272
Smoking: Missing	N/A	N/A	N/A		N/A	N/A	N/A		N/A	N/A	N/A
Alcohol: 1-14	0.69	0.42-1.12	0.1334		0.90	0.52-1.56	0.7099		0.26	0.08-0.84	0.0244
Alcohol: 15-42	0.55	0.17-1.75	0.3119		0.69	0.25-1.92	0.4773		3.61	1.13-11.50	0.0301
Alcohol: >42	1.06	0.15-7.64	0.9539		1.91	0.46-7.83	0.3714		N/A	N/A	N/A
Alcohol: Missing	1.31	0.68-2.51	0.4192		1.37	0.65-2.91	0.4108		0.59	0.08-4.53	0.6141
Morbidities	1.40	1.24-1.58	<0.0001		1.53	1.34-1.74	<0.0001		1.66	1.29-2.13	0.0001
Prescriptions	1.01	0.99-1.03	0.2366		1.02	1.01-1.04	0.0105		1.03	0.99-1.07	0.1831
Hospitalisations	1.66	1.39-1.99	<0.0001		1.84	1.48-2.29	<0.0001		2.12	1.61-2.80	<0.0001
Duration of diabetes (years)	1.02	0.98-1.06	0.3452		1.03	0.99-1.07	0.2017		1.03	0.94-1.12	0.5221
Complications	2.54	2.13-3.02	<0.0001		2.55	2.11-3.07	<0.0001		2.60	1.84-3.68	<0.0001
Glucose lowering therapies	0.88	0.67-1.14	0.3307		1.21	0.91-1.61	0.1829		1.52	0.93-2.50	0.0971
Insulin prescription	4.13	2.51-6.77	<0.0001		4.01	2.14-7.51	<0.0001		6.61	2.22-19.73	0.0007

Study sizes across exposures after 1:1 propensity score matching are provided in Supplementary Table 18, as they are the same between univariate and multivariate analyses.

N/A indicates no observations for the covariate after propensity score matching.

Supplementary Table 18: Multivariate hazard ratios (with corresponding 95% CIs and p-values) for major-only amputation risk by each covariate across QOF exposure definitions, *among those who meet all other QOF targets*, after 1:1 propensity score matching, including the adjusted study size (n) and C-statistic (also with corresponding 95% CI).

	**Exposure Definition**								
	**Achieve HbA1c QOF Target**			**Achieve Blood Pressure QOF Target**			**Achieve Cholesterol QOF Target**		
	**Hazard Ratio**	**95% CI**	**p**	**Hazard Ratio**	**95% CI**	**p**	**Hazard Ratio**	**95% CI**	**p**
Exposure	1.16	0.70-1.92	0.5575	0.89	0.51-1.54	0.6823	0.42	0.14-1.26	0.1205
Age	0.99	0.97-1.02	0.6263	1.02	0.99-1.06	0.2243	1.01	0.96-1.07	0.5996
Sex: Female	0.90	0.50-1.62	0.7308	1.17	0.62-2.21	0.6233	0.82	0.28-2.36	0.7085
Ethnicity: Asian	0.13	0.02-0.94	0.0439	0.72	0.16-3.19	0.6626	N/A	N/A	N/A
Ethnicity: Black	0.58	0.07-4.51	0.6028	N/A	N/A	N/A	N/A	N/A	N/A
Ethnicity: Mixed	2.51	0.34-18.59	0.3674	N/A	N/A	N/A	N/A	N/A	N/A
Ethnicity: Other	1.23	0.16-9.21	0.8404	N/A	N/A	N/A	N/A	N/A	N/A
IMD	1.00	0.96-1.05	0.8995	0.98	0.93-1.03	0.3684	0.97	0.88-1.07	0.5556
North West	1.24	0.16-9.89	0.8363	1.58	0.21-12.10	0.6616	N/A	N/A	N/A
Yorkshire & Humber	1.79	0.18-17.49	0.6158	0.53	0.03-8.55	0.6525	N/A	N/A	N/A
East Midlands	0.97	0.06-16.09	0.9842	N/A	N/A	N/A	N/A	N/A	N/A
West Midlands	0.90	0.10-7.77	0.9207	0.95	0.11-8.22	0.9640	N/A	N/A	N/A
East of England	1.05	0.12-9.14	0.9664	0.91	0.10-8.25	0.9301	N/A	N/A	N/A
South West	2.41	0.31-18.58	0.3994	0.98	0.12-8.10	0.9859	N/A	N/A	N/A
South Central	1.51	0.18-12.39	0.7011	0.54	0.05-5.29	0.5961	N/A	N/A	N/A
London	1.09	0.13-9.15	0.9355	0.69	0.08-6.18	0.7362	N/A	N/A	N/A
South East Coast	2.12	0.27-16.76	0.4781	2.33	0.30-18.15	0.4206	N/A	N/A	N/A
BMI: Underweight	0.48	0.06-3.77	0.4867	0.58	0.08-4.42	0.5953	N/A	N/A	N/A
BMI: Overweight	0.28	0.04-2.16	0.2241	0.34	0.04-2.54	0.2903	N/A	N/A	N/A
BMI: Obese	0.23	0.03-1.74	0.1548	0.15	0.02-1.19	0.0732	N/A	N/A	N/A
BMI: Missing	1.80	0.18-17.64	0.6129	N/A	N/A	N/A	N/A	N/A	N/A
Ex-Smoker	0.98	0.55-1.76	0.9426	2.49	1.23-5.02	0.0110	1.15	0.40-3.33	0.7908
Current Smoker	1.71	0.83-3.50	0.1429	2.53	0.99-6.44	0.0522	3.61	1.13-11.52	0.0300
Smoking: Missing	N/A	N/A	N/A	N/A	N/A	N/A	N/A	N/A	N/A
Alcohol: 1-14	0.54	0.29-0.98	0.0436	0.98	0.44-2.17	0.9645	0.22	0.07-0.71	0.0111
Alcohol: 15-42	0.41	0.12-1.43	0.1607	0.81	0.23-2.79	0.7324	1.16	0.36-3.72	0.8081
Alcohol: >42	0.41	0.05-3.24	0.3963	2.37	0.49-11.58	0.2847	N/A	N/A	N/A
Alcohol: Missing	1.25	0.58-2.69	0.5750	1.83	0.68-4.94	0.2314	0.33	0.04-2.53	0.2867
Morbidities	0.92	0.78-1.09	0.3263	1.02	0.85-1.22	0.8662	1.11	0.88-1.40	0.3933
Prescriptions	1.00	0.97-1.03	0.8455	1.00	0.97-1.03	0.9660	0.97	0.91-1.03	0.3046
Hospitalisations	1.34	1.06-1.69	0.0152	1.37	1.01-1.86	0.0445	1.87	1.36-2.56	0.0001
Duration of diabetes (years)	1.00	0.96-1.04	0.9825	1.01	0.96-1.06	0.6850	1.02	0.93-1.11	0.7388
Complications	2.50	2.02-3.09	<0.0001	2.14	1.69-2.70	<0.0001	2.10	1.47-2.99	<0.0001
Glucose lowering therapies	0.82	0.59-1.14	0.2314	1.13	0.79-1.62	0.5018	1.37	0.81-2.29	0.2375
Insulin prescription	2.60	1.51-4.47	0.0005	2.33	1.14-4.78	0.0205	2.46	0.82-7.35	0.1077

	**Value**	**95% CI (lower)**	**95% CI (upper)**		**Value**	**95% CI (lower)**	**95% CI (upper)**		**Value**	**95% CI (lower)**	**95% CI (upper)**
n after matching	20,462				32,300				11,756		
C-statistic	0.8553	0.8539	0.8568		0.8907	0.8898	0.8915		0.9493	0.9481	0.9506

Reference groups for categorical covariates include: white (ethnicity); North East (region); normal weight (BMI); non-smoker (smoking status); 0 units (alcohol consumption); and no insulin prescription (insulin use).

N/A indicates no observations for the covariate after propensity score matching.

Supplementary Table 19: Univariate hazard ratios (with corresponding 95% CIs and p-values) for major-only amputation risk by each covariate across QOF exposure definitions, *among those who do not meet either of the other two QOF targets*, after 1:1 propensity score matching.

	Exposure Definition										
	Achieve HbA1c QOF Target				Achieve Blood Pressure QOF Target				Achieve Cholesterol QOF Target		
	Hazard Ratio	95% CI	p		Hazard Ratio	95% CI	p		Hazard Ratio	95% CI	p
Exposure	0.24	0.07-0.85	0.0269		0.78	0.39-1.56	0.4771		0.89	0.50-1.58	0.6793
Age	1.05	1.01-1.10	0.0167		1.02	0.99-1.05	0.1323		1.02	1.00-1.04	0.0762
Sex: Female	1.03	0.37-2.85	0.9490		0.86	0.43-1.72	0.6648		1.20	0.67-2.14	0.5370
Ethnicity: Asian	N/A	N/A	N/A		0.41	0.06-2.97	0.3738		0.26	0.04-1.85	0.1772
Ethnicity: Black	N/A	N/A	N/A		N/A	N/A	N/A		0.63	0.09-4.55	0.6451
Ethnicity: Mixed	N/A	N/A	N/A		N/A	N/A	N/A		N/A	N/A	N/A
Ethnicity: Other	N/A	N/A	N/A		N/A	N/A	N/A		N/A	N/A	N/A
IMD	1.04	0.95-1.15	0.3640		1.02	0.96-1.08	0.5792		1.03	0.98-1.09	0.2534
North West	3.13	1.12-8.80	0.0302		1.35	0.58-3.12	0.4828		2.02	1.06-3.84	0.0318
Yorkshire & Humber	N/A	N/A	N/A		N/A	N/A	N/A		N/A	N/A	N/A
East Midlands	3.46	0.45-26.36	0.2304		2.82	0.67-11.80	0.1559		1.09	0.15-7.91	0.9314
West Midlands	0.54	0.07-4.14	0.5567		1.62	0.67-3.94	0.2866		1.04	0.44-2.46	0.9257
East of England	N/A	N/A	N/A		0.61	0.14-2.54	0.4933		0.86	0.31-2.39	0.7709
South West	1.56	0.44-5.51	0.4939		1.80	0.78-4.16	0.1696		0.95	0.40-2.24	0.9039
South Central	1.12	0.25-4.98	0.8771		0.50	0.12-2.07	0.3363		0.47	0.14-1.50	0.2004
London	0.46	0.06-3.47	0.4492		0.43	0.10-1.81	0.2507		1.00	0.43-2.37	0.9924
South East Coast	0.49	0.06-3.74	0.4924		0.66	0.20-2.16	0.4924		1.18	0.53-2.64	0.6832
BMI: Underweight	1.18	0.27-5.25	0.8245		1.89	0.82-4.36	0.1378		0.45	0.11-1.85	0.2680
BMI: Overweight	1.17	0.40-3.41	0.7787		0.72	0.32-1.60	0.4225		0.71	0.35-1.43	0.3364
BMI: Obese	0.87	0.32-2.41	0.7938		0.99	0.49-1.98	0.9710		1.83	0.95-3.53	0.0728
BMI: Missing	N/A	N/A	N/A		N/A	N/A	N/A		N/A	N/A	N/A
Ex-Smoker	1.38	0.49-3.87	0.5444		1.67	0.83-3.36	0.1503		0.96	0.52-1.78	0.8949
Current Smoker	1.96	0.62-6.15	0.2501		0.95	0.39-2.30	0.9018		2.48	1.34-4.59	0.0039
Smoking: Missing	N/A	N/A	N/A		N/A	N/A	N/A		N/A	N/A	N/A
Alcohol: 1-14	0.68	0.25-1.89	0.4629		1.11	0.55-2.25	0.7666		0.68	0.38-1.21	0.1853
Alcohol: 15-42	N/A	N/A	N/A		0.35	0.05-2.56	0.3004		0.20	0.03-1.47	0.1147
Alcohol: >42	2.16	0.28-16.42	0.4573		N/A	N/A	N/A		1.91	0.46-7.87	0.3717
Alcohol: Missing	1.59	0.45-5.63	0.4733		1.30	0.54-3.16	0.5606		1.91	0.99-3.68	0.0547
Morbidities	1.64	1.29-2.10	0.0001		1.18	0.97-1.43	0.1035		1.39	1.19-1.62	<0.0001
Prescriptions	0.99	0.92-1.06	0.7821		0.99	0.96-1.03	0.7991		1.01	0.99-1.04	0.2688
Hospitalisations	1.03	0.37-2.88	0.9509		1.29	0.85-1.96	0.2307		1.38	1.06-1.81	0.0173
Duration of diabetes (years)	1.00	0.91-1.11	0.9287		1.00	0.94-1.07	0.9703		1.01	0.97-1.07	0.5631
Complications	3.32	2.34-4.71	<0.0001		2.71	2.13-3.46	<0.0001		3.01	2.44-3.71	<0.0001
Glucose lowering therapies	0.68	0.36-1.26	0.2208		1.39	1.01-1.93	0.0452		0.93	0.69-1.25	0.6379
Insulin prescription	1.85	0.42-8.21	0.4173		2.44	1.21-4.95	0.0131		2.87	1.59-5.15	0.0004

Study sizes across exposures after 1:1 propensity score matching are provided in Supplementary Table 20, as they are the same between univariate and multivariate analyses.

N/A indicates no observations for the covariate after propensity score matching.

Supplementary Table 20: Multivariate hazard ratios (with 95% CIs and p-values) for major-only amputation risk by covariates across QOF exposures, *among those who do not meet either of the other two targets*, after 1:1 propensity score matching, including the adjusted study size (n) and C-statistic (also with 95% CI).

	**Exposure Definition**								
	**Achieve HbA1c QOF Target**			**Achieve Blood Pressure QOF Target**			**Achieve Cholesterol QOF Target**		
	**Hazard Ratio**	**95% CI**	**p**	**Hazard Ratio**	**95% CI**	**p**	**Hazard Ratio**	**95% CI**	**p**
Exposure	0.19	0.05-0.73	0.0154	0.85	0.41-1.75	0.6615	0.85	0.47-1.54	0.5902
Age	1.02	0.97-1.08	0.4465	0.99	0.96-1.03	0.6364	0.99	0.96-1.02	0.4647
Sex: Female	1.57	0.44-5.58	0.4861	1.64	0.73-3.71	0.2322	1.74	0.89-3.39	0.1044
Ethnicity: Asian	N/A	N/A	N/A	0.36	0.05-2.80	0.3264	0.29	0.04-2.19	0.2279
Ethnicity: Black	N/A	N/A	N/A	N/A	N/A	N/A	0.79	0.11-5.88	0.8180
Ethnicity: Mixed	N/A	N/A	N/A	N/A	N/A	N/A	N/A	N/A	N/A
Ethnicity: Other	N/A	N/A	N/A	N/A	N/A	N/A	N/A	N/A	N/A
IMD	1.03	0.92-1.14	0.6563	1.01	0.94-1.08	0.8231	1.02	0.96-1.08	0.4836
North West	N/A	N/A	N/A	1.36	0.16-11.67	0.7785	N/A	N/A	N/A
Yorkshire & Humber	N/A	N/A	N/A	N/A	N/A	N/A	N/A	N/A	N/A
East Midlands	N/A	N/A	N/A	2.42	0.21-27.83	0.4776	N/A	N/A	N/A
West Midlands	N/A	N/A	N/A	1.72	0.19-15.40	0.6265	N/A	N/A	N/A
East of England	N/A	N/A	N/A	0.49	0.04-5.93	0.5738	N/A	N/A	N/A
South West	N/A	N/A	N/A	1.26	0.15-10.89	0.8308	N/A	N/A	N/A
South Central	N/A	N/A	N/A	0.49	0.04-5.94	0.5792	N/A	N/A	N/A
London	N/A	N/A	N/A	0.43	0.04-4.93	0.4948	N/A	N/A	N/A
South East Coast	N/A	N/A	N/A	0.70	0.07-7.31	0.7693	N/A	N/A	N/A
BMI: Underweight	N/A	N/A	N/A	N/A	N/A	N/A	N/A	N/A	N/A
BMI: Overweight	N/A	N/A	N/A	N/A	N/A	N/A	N/A	N/A	N/A
BMI: Obese	N/A	N/A	N/A	N/A	N/A	N/A	N/A	N/A	N/A
BMI: Missing	N/A	N/A	N/A	N/A	N/A	N/A	N/A	N/A	N/A
Ex-Smoker	1.13	0.32-4.00	0.8525	1.63	0.70-3.79	0.2562	0.92	0.44-1.91	0.8203
Current Smoker	2.21	0.52-9.36	0.2834	0.94	0.32-2.76	0.9158	2.19	1.03-4.68	0.0428
Smoking: Missing	N/A	N/A	N/A	N/A	N/A	N/A	N/A	N/A	N/A
Alcohol: 1-14	0.46	0.12-1.71	0.2456	0.96	0.36-2.51	0.9271	0.78	0.36-1.73	0.5456
Alcohol: 15-42	N/A	N/A	N/A	0.40	0.05-3.57	0.4143	0.19	0.02-1.57	0.1231
Alcohol: >42	1.50	0.13-17.44	0.7476	N/A	N/A	N/A	1.17	0.22-6.09	0.8532
Alcohol: Missing	1.07	0.21-5.44	0.9388	1.50	0.47-4.80	0.4914	1.84	0.77-4.36	0.1674
Morbidities	1.08	0.77-1.51	0.6545	0.81	0.63-1.04	0.0917	0.88	0.73-1.07	0.2049
Prescriptions	0.99	0.92-1.07	0.7991	0.94	0.87-1.01	0.0697	1.00	0.96-1.03	0.8632
Hospitalisations	0.56	0.18-1.72	0.3078	0.96	0.60-1.55	0.8719	0.96	0.66-1.39	0.8302
Duration of diabetes (years)	0.95	0.85-1.06	0.3535	0.98	0.91-1.05	0.5968	1.01	0.96-1.06	0.7255
Complications	3.54	2.23-5.64	<0.0001	3.32	2.46-4.49	<0.0001	3.41	2.64-4.40	<0.0001
Glucose lowering therapies	0.53	0.24-1.18	0.1216	1.57	1.00-2.46	0.0497	0.79	0.55-1.14	0.2117
Insulin prescription	0.75	0.13-4.32	0.7453	1.00	0.44-2.27	0.9965	0.92	0.46-1.83	0.8031
	**Value**	**95% CI (lower)**	**95% CI (upper)**	**Value**	**95% CI (lower)**	**95% CI (upper)**	**Value**	**95% CI (lower)**	**95% CI (upper)**
n after matching	8,286			8,222			9,752		
C-statistic	0.9591	0.9587	0.9596	0.9116	0.9103	0.9129	0.9229	0.9219	0.9239

Reference groups for categorical covariates include: white (ethnicity); North East (region); normal weight (BMI); non-smoker (smoking status); 0 units (alcohol consumption); and no insulin prescription (insulin use).

N/A indicates no observations for the covariate after propensity score matching.

Supplementary Figure 4: Kaplan-Meier survival curves (and corresponding 95% CIs) for major-only amputation risk after 1:1 propensity score matching across exposure definitions.

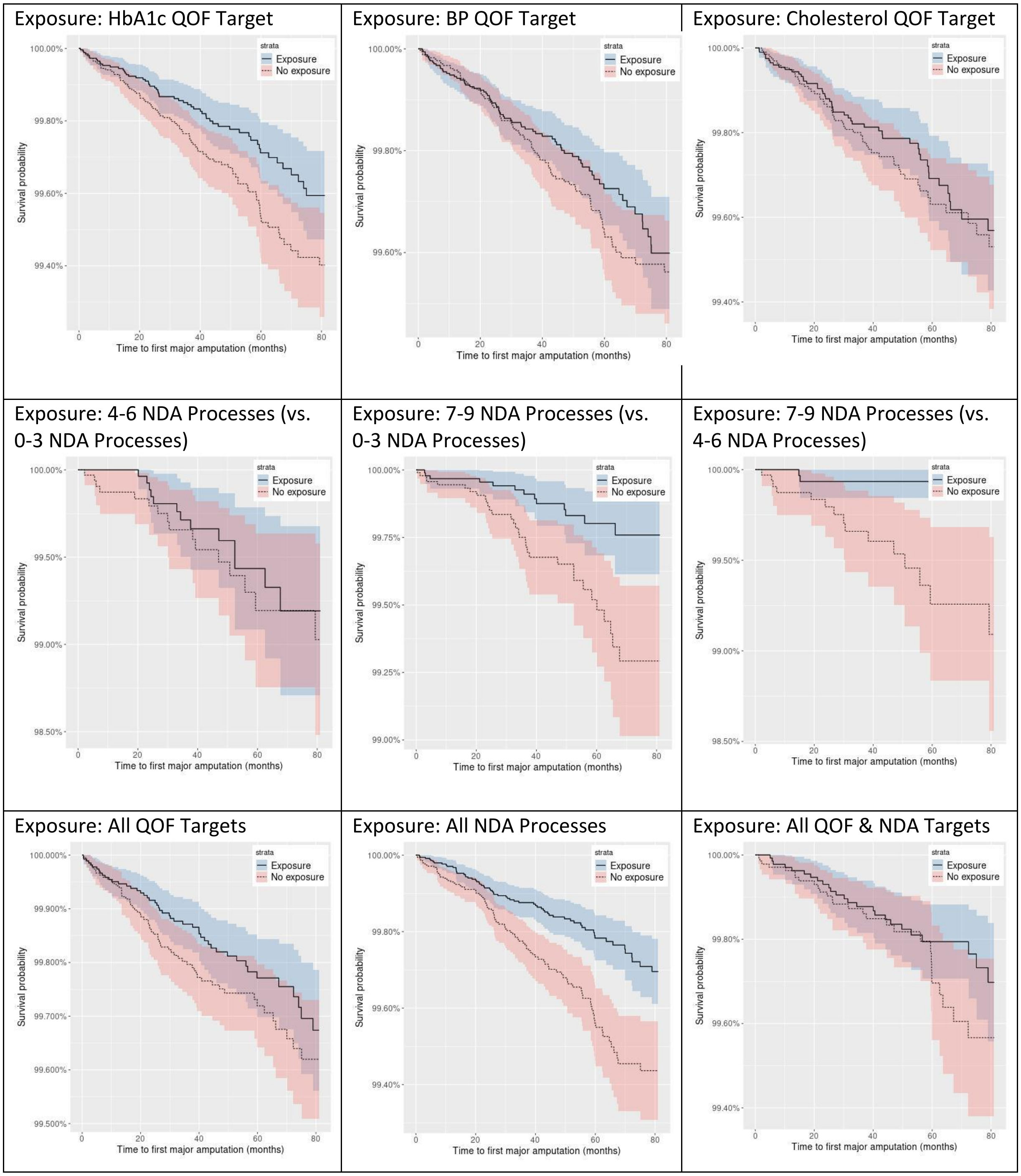

Supplementary Figure 5: Kaplan-Meier survival curves (and corresponding 95% CIs) for major-only amputation risk after 1:1 propensity score matching across QOF exposure definitions, *among those who meet all other QOF targets*.

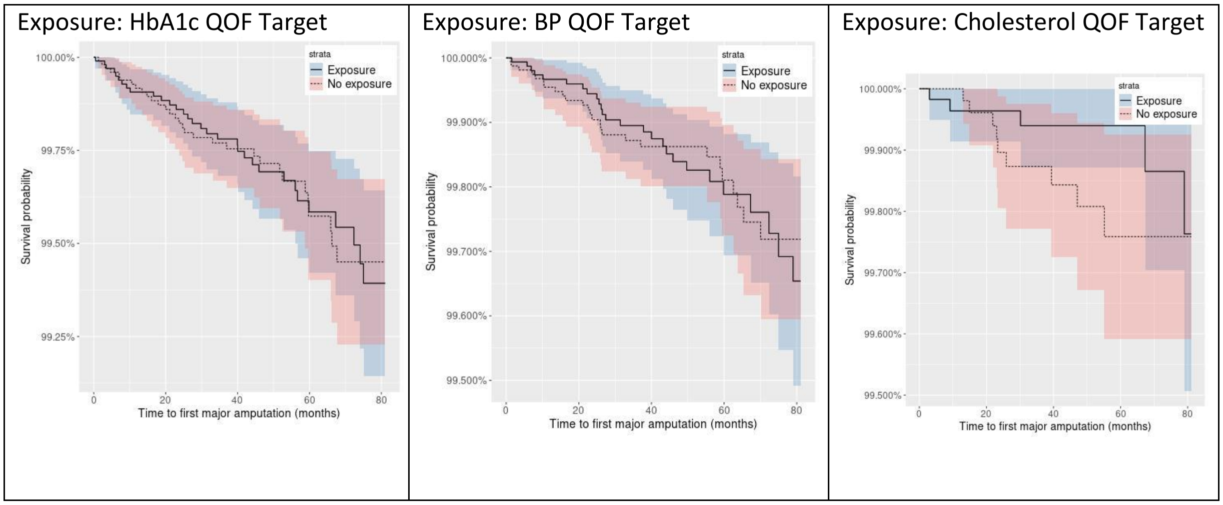

Supplementary Figure 6: Kaplan-Meier survival curves (and corresponding 95% CIs) for major-only amputation risk after 1:1 propensity score matching across QOF exposure definitions, *among those who do not meet either of the other two QOF targets*.

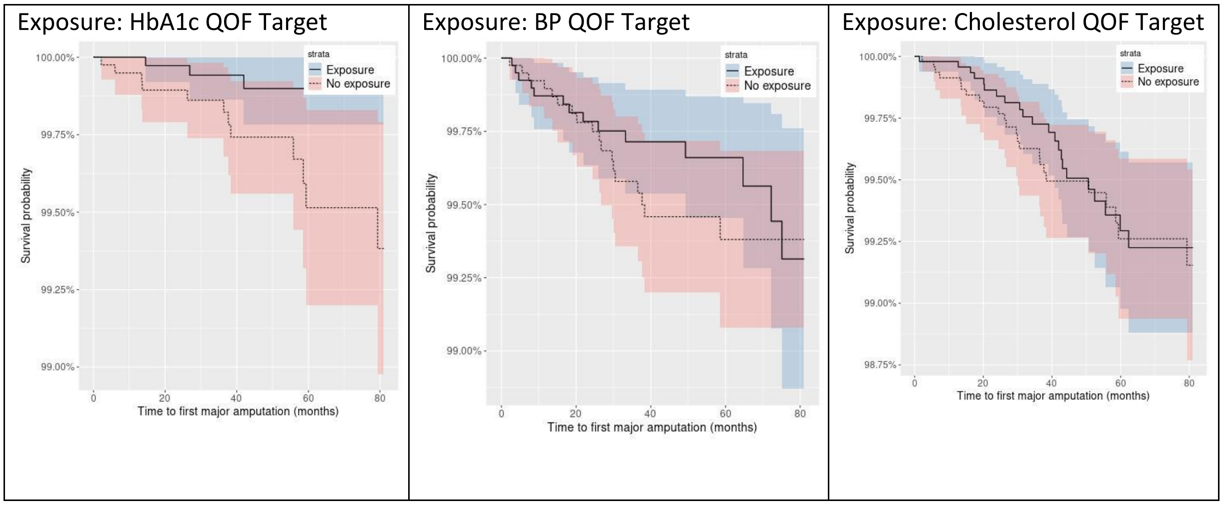

Supplement: Supplementary data [file bmjdrc-2020-002069supp001.pdf]
